# Unveiling the interplay between mutational signatures and tumor microenvironment: a pan-cancer analysis

**DOI:** 10.3389/fimmu.2023.1186357

**Published:** 2023-05-22

**Authors:** Li-Zhi Luo, Sheng Li, Chen Wei, Jiao Ma, Li-Mei Qian, Yan-Xing Chen, Shi-Xiang Wang, Qi Zhao

**Affiliations:** ^1^ State Key Laboratory of Oncology in South China, Collaborative Innovation Center for Cancer Medicine, Sun Yat-Sen University Cancer Center, Sun Yat-Sen University, Guangzhou, China; ^2^ School of Public Health, Sun Yat-Sen University, Guangzhou, China

**Keywords:** mutational signatures, tumor microenvironment, cancer genomics, cancer prognosis, immunotherapy

## Abstract

**Background:**

While recent studies have separately explored mutational signatures and the tumor microenvironment (TME), there is limited research on the associations of both factors in a pan-cancer context.

**Materials and methods:**

We performed a pan-cancer analysis of over 8,000 tumor samples from The Cancer Genome Atlas (TCGA) project. Machine learning methods were employed to systematically explore the relationship between mutational signatures and TME and develop a risk score based on TME-associated mutational signatures to predict patient survival outcomes. We also constructed an interaction model to explore how mutational signatures and TME interact and influence cancer prognosis.

**Results:**

Our analysis revealed a varied association between mutational signatures and TME, with the Clock-like signature showing the most widespread influence. Risk scores based on mutational signatures mainly induced by Clock-like and AID/APOBEC activity exhibited strong pan-cancer survival stratification ability. We also propose a novel approach to predict transcriptome decomposed infiltration levels using genome-derived mutational signatures as an alternative approach for exploring TME cell types when transcriptome data are unavailable. Our comprehensive analysis revealed that certain mutational signatures and their interaction with immune cells significantly impact clinical outcomes in particular cancer types. For instance, T cell infiltration levels only served as a prognostic biomarker in melanoma patients with high ultraviolet radiation exposure, breast cancer patients with high homologous recombination deficiency signature, and lung adenocarcinoma patients with high tobacco-associated mutational signature.

**Conclusion:**

Our study comprehensively explains the complex interplay between mutational signatures and immune infiltration in cancer. The results highlight the importance of considering both mutational signatures and immune phenotypes in cancer research and their significant implications for developing personalized cancer treatments and more effective immunotherapy.

## Introduction

Mutational signatures refer to the specific patterns of mutations arising in cancer genomes due to DNA damage from endogenous and exogenous sources (1, 2). They have become increasingly important in understanding the underlying mechanisms of carcinogenesis and have been extensively studied to gain insight into various factors contributing to cancer development (3–6). The significance of mutational signatures in cancer research extends to both basic and clinical studies, providing important information for developing targeted therapies and improving patient outcomes (7, 8). Studies on mutational signatures, such as those related to homologous recombination repair defects (9–12), mismatch repair defects (13, 14), and AID/APOBEC mutagenesis (15–17), have significant implications for the progress of precision medicine and immunotherapy in cancer. These mutational signatures provide valuable insights into the underlying mechanisms of tumorigenesis, allowing for more informed therapeutic decisions based on a patient’s specific genetic landscape. By identifying and targeting specific mutational signatures, researchers can develop tailored treatments optimized for the unique genetic characteristics of an individual patient’s tumor.

The tumor microenvironment (TME) is an essential aspect of cancer biology that involves the interactions of various components within the microenvironment, such as tumor cells, immune cells, stromal cells, and the extracellular matrix (18, 19). The composition and functions of the TME have a significant impact on tumor growth and progression, as well as cancer treatment efficacy (20–22). Recent studies have demonstrated the crucial role of the TME in regulating anti-tumor immunity and shaping responses to cancer therapies (23, 24). In particular, the presence of immune cells, such as T cells and immune checkpoint molecules, and their interactions with tumor cells and the surrounding stroma can either inhibit or promote tumor progression and influence the response to cancer treatments (25–27). As a result, TME has become a promising target for cancer therapy and is attracting increasing attention from the scientific community.

The relationship between mutational signatures and the TME remains largely unknown, despite their growing recognition and importance. Therefore, investigating the interplay between mutational signatures and the TME is crucial to improve current cancer diagnosis and treatment approaches, including the discovery of novel checkpoint immunotherapy biomarkers (28), the transformation of non-responsive “cold” tumors into responsive “hot” tumors (29), and the development of strategies to overcome resistance to checkpoint immunotherapy (30) and T cell-based immunotherapy (31). To address this knowledge gap, this present study aims to comprehensively analyze the interplay between mutational signatures and the tumor immune microenvironment via pan-cancer analysis. The primary aim of this analysis is to identify the immune features and cell types influenced by specific mutational signatures. Given the pivotal role of this interaction in determining the clinical outcome of tumors, our study is expected to improve our understanding of the mutational processes involved in tumor immune response and help advance the field of precision cancer therapy.

## Results

### Overview of pan-cancer mutational signatures and the tumor microenvironment

**Figure 1:**
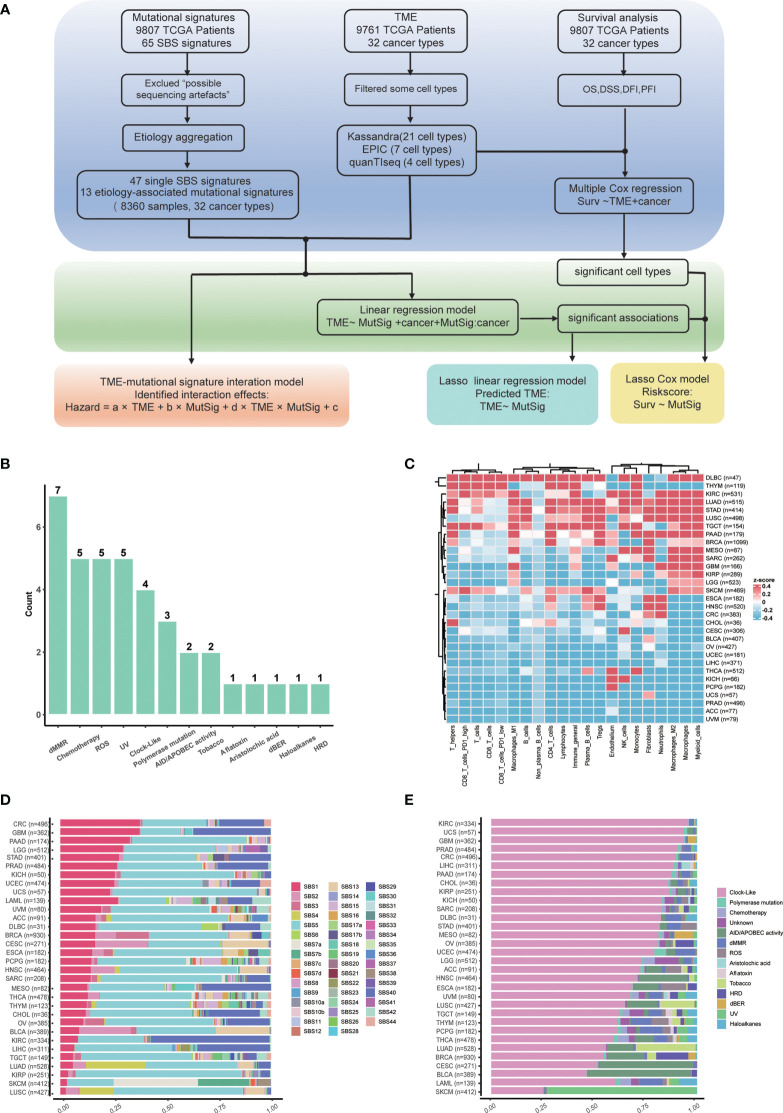
Study flowchart and data overview. **(A)** Flowchart of this study. **(B)** Summary of etiology-associated mutational signatures. The number of SBS mutational signatures combined with each etiology has been shown as the number above the bar. Detailed data are available in **Supplementary Table 1**. **(C)** Abundances heatmap of 21 cell types in the TME across cancer types based on the Kassandra approach. **(D)** Averaged proportions of SBS mutational signatures across cancer types. The mutational signatures are sorted according to the proportion of SBS1. **(E)** Averaged proportions of etiology-associated mutational signatures across cancer types. The mutational signatures are sorted according to the proportion of Clock-like-associated mutational signatures.

Overview of our study is shown in **Figure 1A**. In total, 47 non-artificial single base substitution (SBS) mutational signatures annotated in the COSMIC mutational signature database (https://cancer.sanger.ac.uk/signatures/sbs/) are included. These mutational signatures were further aggregated as 13 types of “etiology-associated” mutational signatures to facilitate the biological interpretation of these signatures. In this process, seven SBS mutational signatures with annotation related to defects in mismatch repair (dMMR) were merged into the dMMR-associated mutational signature (**Figure 1B**). In comparison, only SBS3 is related to homologous recombination deficiency (HRD), therefore we just renamed SBS3 as HRD-associated mutational signature. To accurately describe the tumor microenvironment, we adopted the data of 21 cell types from the state-of-the-art approach Kassandra (32). We observed a heterogeneous distribution of tumor immune infiltration, with cancer type DLBC having the most inflamed tumor microenvironment while UVM having the most non-inflamed phenotype (**Figure 1C**). This observation is consistent with previous reports (33).

Next, we examined the pan-cancer level abundance of mutational signatures in SBS mutational signature or etiology-associated mutational signature manner, respectively (**Figures 1D, E**). We found that the constitution of mutational signatures varied greatly across tumor types. For instance, SBS1 activity decreased from 40% in colorectal cancer (CRC) to 5% in lung squamous cell carcinoma (LUSC) (**Figure 1D**). Similar patterns were observed for the etiology-associated mutational signature “Clock-like”, indicating that aging contributes significantly to the tumor mutation load and the history of tumor formation and development (**Figure 1E**) (34). Meanwhile, we also identified cancer type-specific mutational signatures such as UV (ultraviolet radiation exposure) in skin cutaneous melanoma (SKCM) and Tobacco in lung adenocarcinoma (LUAD) and lung squamous cell carcinoma (LUSC). Interestingly, the dMMR-associated mutational signature accounted for approximately 20% of colorectal cancer, which could explain the presence of MSI-H in around 15% of colorectal cancer (35). The dMMR-associated signature was also active in pancreatic cancer and diffuse large B cell lymphoma, suggesting that it may be a valuable immunotherapy marker in these cancers. Conversely, kidney cancer, nasopharyngeal carcinoma, as well as head and neck squamous cell carcinoma have low dMMR proportions (36), suggesting that the dMMR signature may not be a suitable immunotherapy marker for these cancers.

### Panorama of interrelation between mutational signatures and the tumor microenvironment

**Figure 2:**
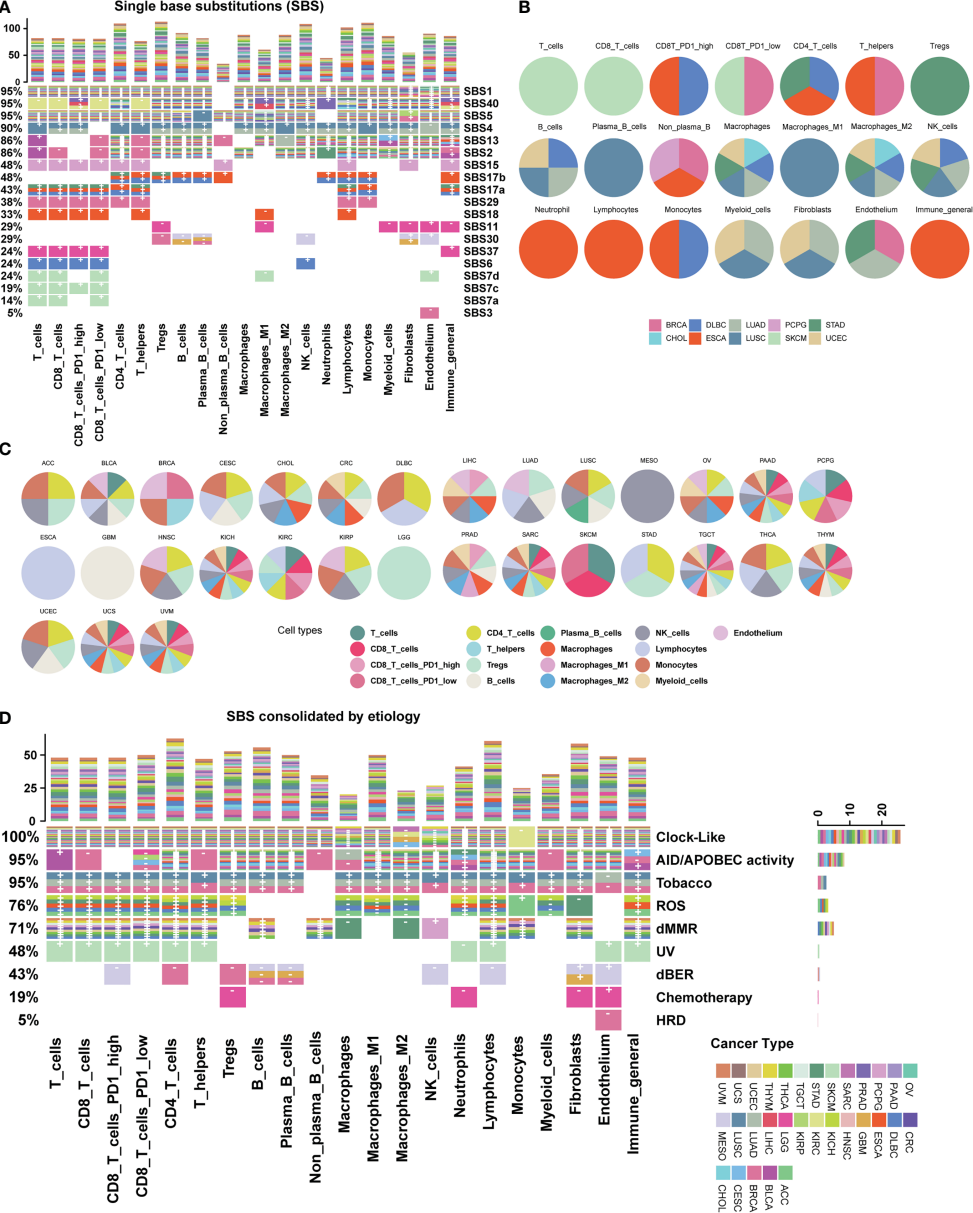
Association map between mutational signatures and cell types in the tumor microenvironment. **(A)** Heatmap depicting the relationship between SBS mutational signatures and TME cell types in 31 cancer types. Each row represents a SBS mutational signature, and each color represents a cancer type. The percentage on the left represents the proportion of cell types affected by each SBS mutational signature. The bar chart at the top shows the number of associations with mutational signatures per cell type. “+” and “-” are signs for positive association and negative association, respectively. Color categories indicate cancer types where the association has been found. Blank squares indicate that a SBS mutational signature was not found to have an association with a particular immune cell type with a threshold FDR<0.1. Detailed data are available in **Supplementary Table 3**. The result of uncalibrated *P*-values is shown in **Supplementary Figure 1**. **(B)** Top cancer types with the highest number of associations per cell type. **(C)** Top TME cell types with the highest number of associations per cancer type. **(D)** Heatmap depicting the etiology-associated mutational signatures associated with TME cell types in 31 cancer types. Detailed data are available in **Supplementary Tables 3**, **4**.

We further explored the relationship between mutational signatures and the TME and found their broad connections in cancer (**Figure 2**; **Supplementary Figure 1**). Mutational signatures SBS1, SBS2, SBS5, SBS13, SBS40, etc. have extensive pan-cancer associations with immune microenvironments, while SBS4, SBS7, SBS11, SBS15, SBS17, etc. exhibit cancer type-specific property or immune cell type bias. In the meantime, more than half of SBS mutational signatures lack correlation to the TME (**Figure 2A**). We listed the top cancer types for each cell type with the highest number of significantly associated SBS mutational signatures and found that PD1 high CD8 T cell was associated with four SBS mutational signatures in both ESCA and DLBC (**Figures 2A, B**). We also listed the top cell types for each cancer type with the highest number of significantly associated SBS mutational signatures (**Figure 2C**). For example, cell type “lymphocytes” was associated with seven SBS mutational signatures in cancer type ESCA (**Figures 2A, C**). We conducted additional analyses to investigate whether the association between mutational signatures and TME is dependent on age, gender and tumor stage. Our results show that these factors do not seem to significantly impact our findings (**Supplementary Figure 2A, B**).

By analyzing etiology-associated mutational signatures, we identified three distinct connection patterns. The first pattern involves Clock-like and AID/APOBEC activity-associated mutational signatures, which have a pan-cancer impact on diverse TME cell types (**Figure 2D**; **Supplementary Table 3**). In detail, we found that the Clock-like associated mutational signature is associated with more than 26 cancer types across 17 cell types, except for a few cell types such as macrophages M2 (four cancer types), NK (nine cancer types), macrophages (11 cancer types), and monocytes (one cancer type). Similarly, the AID/APOBEC activity associated mutational signatures is associated with 15 cancer types across 20 cell types. The second pattern includes endogenous ROS (reactive oxygen species), dMMR, and dBER (DNA base excision repair) associated mutational signatures, which exhibit associations with multiple TME cell types in a few cancer types. The ROS-associated mutational signature is related to 16 cell types in six cancer types: ACC, DLBC, ESCA, KICH, STAD, and THYM. The dBER-associated mutational signatures are associated with nine cell types in three cancer types: BRCA, GBM, and MESO. The dMMR-associated mutational signatures involves 15 cell types, such as B cells, CD4T cells, CD8T cells, fibroblasts, NK cells, and macrophages, and are associated with nine cancer types: CRC, DLBC, PCPG, STAD, TGCT, THCA, THYM, UCEC, and UVM. The third pattern involves cancer type-specific mutational signatures from environmental or endogenous causes, such as the HRD-associated mutational signatures in BRCA, the Tobacco-associated mutational signatures in lung cancer including LUAD, and LUSC, and UV-associated mutational signatures in SKCM.

The remaining etiology-associated mutational signatures including Polymerase mutation, Aristolochic acid, Aflatoxin and Haloalkanes, show little association with TME cell types under a significance level of FDR < 0.1. Additionally, we validated our data with other two recommended TME decomposition methods EPIC and quanTIseq from TIMER 2.0 database (37, 38), similar associations and patterns are observed (**Supplementary Figures 2C–F**).

### Prediction of immune infiltration levels by mutational signatures in cancer

The tumor microenvironment plays a significant role in tumor evolution and can serve as a predictor for both prognosis and response to immunotherapy (25). In recent years, transcriptome analysis has been widely used to evaluate the infiltration levels of TME cells. However, measurements of the TME cell types such as tumor-infiltrating lymphocytes are limited by a shortage of appropriate data analysis methods, particularly in genomics. In light of the extensive connections between many mutational signatures and TME cell types observed in our data, we hypothesized that mutational signatures derived from cancer genomics could be used to predict immune infiltration levels. To test this hypothesis, we selected mutational signatures significantly associated with TME and employed LASSO (39) to develop a prediction model based on mutational signatures to infer the infiltration levels of TME cell types across cancer types.

**Figure 3:**
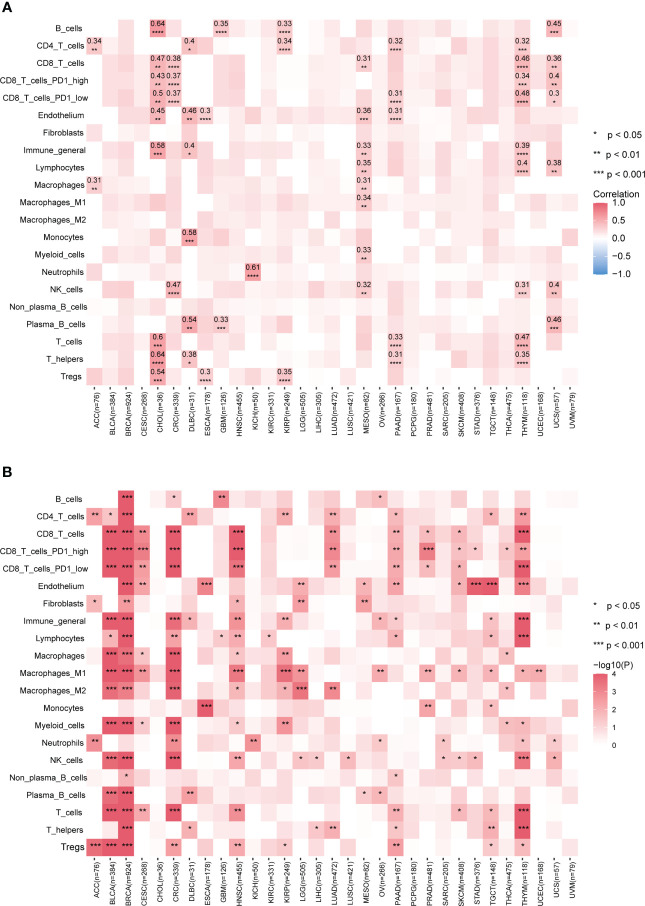
Performance of predicting tumor immune infiltration with LASSO regression modeling on mutational signatures. **(A)** Spearman correlation of predicted and true infiltration values for different cell types. Only absolute Spearman correlation coefficients >0.3 have been shown. Blank boxes indicate that the constructed model could not predict the cell type. **(B)** Evaluation of the status of actual infiltration by grouping the predicted values. Wilcoxon test, * p<0.05, ** p<0.01, *** p<0.001, **** p<0.0001. More results are provided in **Supplementary Figure 3**. Detailed data are available in **Supplementary Table 7**.

Our results show that the prediction performance of our model varied across different cancer types and TME cell types (**Figure 3A**). Specifically, for B cells, T cells, and T helper cells in cancer type CHOL and neutrophil cells in cancer type KICH, the correlation coefficients between predicted values and Kassandra inferred values were above 0.6; for T helpers, plasma B cells, monocytes, CD4T cells, endothelium, and T cells (including CD8T cells with low PD1 expression) in THYM, as well as B cells and plasma B cells in UCS, the correlation coefficients can still reach a level of 0.4-0.5. Besides, our model has the statistically significant prediction ability for inferring the infiltration levels of CD8 T cells in cancer types including CHOL, CRC, MESO, THYM and UCS instead of BLCA, BRCA, etc. This is confirmed by using TME decomposition methods EPIC and quanTIseq (**Supplementary Figure 3**).

Although the poor prediction performance in BLCA and BRCA, our further analysis found that classification based on the predicted infiltration levels of most TME cell types could efficiently distinguish the abundance distribution of corresponding cell types (**Figure 3B**; **Supplementary Figure 3**). This reflects mutational signatures tend to indirectly alter the status of the tumor immune microenvironment, rather than directly causing biological coordinated changes. Altogether, our data suggest that our prediction model based on mutational signature to some extent could be a surrogate approach when no transcriptomic data available.

### Pan-cancer prognosis stratification with immune-related mutational signatures

**Figure 4:**
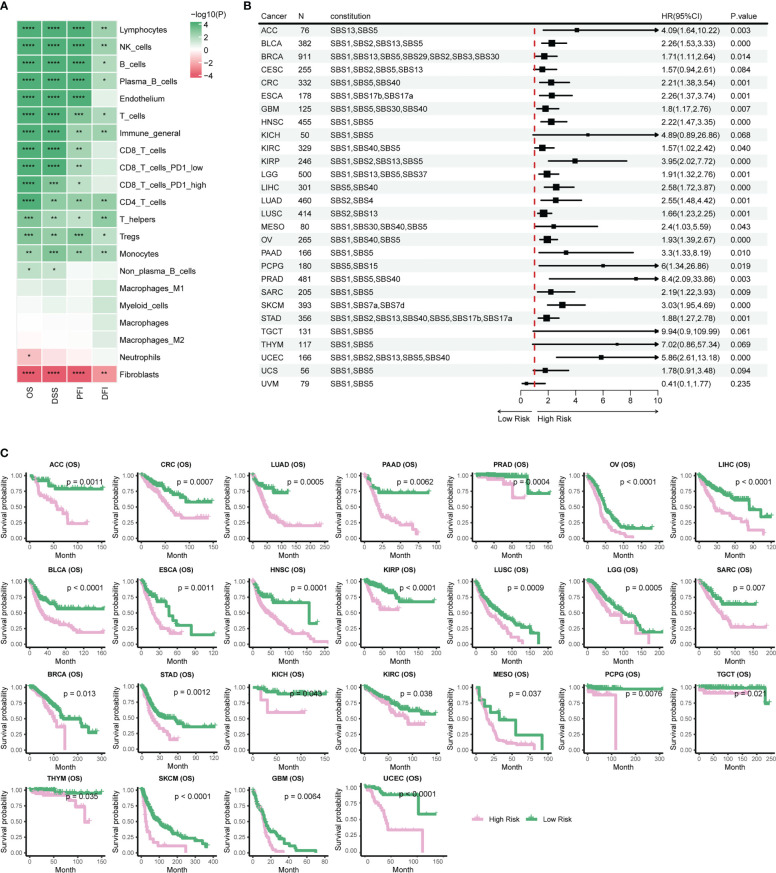
Stratification of patient survival based on immune-related SBS mutational signatures. **(A)** TME cell types associated with pan-cancer survival. Hazard ratio (HR) values ranging from 0 to 1 are colored in green and represent protective factors, while HR values exceeding 1 are colored in red and represent risk factors. **(B)** Forest plot displaying the associations between immune-related mutational signatures derived risk scores and patient overall survival using univariate Cox regression analysis. **(C)** Kaplan–Meier analysis for patients with high and low risk scores in different cancer types. High-risk scores are associated with generally poor survival. Detailed data are available in **Supplementary Table 8**. #*p<0.05, ** p<0.01, *** <0.001, **** p<0.0001.

Prognostic biomarkers enable identification of patients with a more aggressive tumor evolution (40). Here we first investigate the prognostic significance of various immune cells by assessing four survival endpoints (OS, overall survival; PFI, progression free interval; DFI, disease free interval; DSS, disease specific survival) in multiple cancer types. Our data show that CD8 T cells and lymphocytes are protective factors, while cancer-associated fibroblasts (CAFs) acts as a risk factor, consistent with previous report (**Figure 4A**) (18). To further explore the prognosis significance of mutational signatures related to the immune infiltration, we established an overall survival risk score using the LASSO Cox model. We then evaluated the prognostic performance of the risk score in various cancer types (**Figures 4B, C**). Notably, our risk score exhibits effective discrimination in 25 out of 31 cancer types. Although the tests for CES and UCS are insignificant, they still showed a stratification trend (**Supplementary Figures 4B, C**).

Risk scores of most cancer types consists of contribution from multiple mutational signatures, including the Clock-like signature (SBS1, SBS5, and SBS40) and the AID/APOBEC activity signature (SBS2 and SBS13) (**Figure 4B**). This is consistent with previous studies showing that Clock-like and AID/APOBEC activity signatures could significantly affect tumor growth and patient prognosis (34, 41). Moreover, our etiology-associated mutational signatures analysis further demonstrated that the Clock-like signature and the AID/APOBEC activity signature are the major contributors of risk scores in many types of cancer, with additional mutational signatures contributing to some specific cancer types, such as dMMR in CRC and HRD in BRCA. These findings align with prognosis analyses of previous studies for dMMR or MSI-H in CRC and HRD in BRCA (9–14). In summary, our data support a novel risk score based on immune-related mutational signatures for pan-cancer prognosis stratification, which may have important implications for personalized cancer therapy.

### A statistical interaction test identifies mutational signatures of immune phenotype alteration

**Figure 5:**
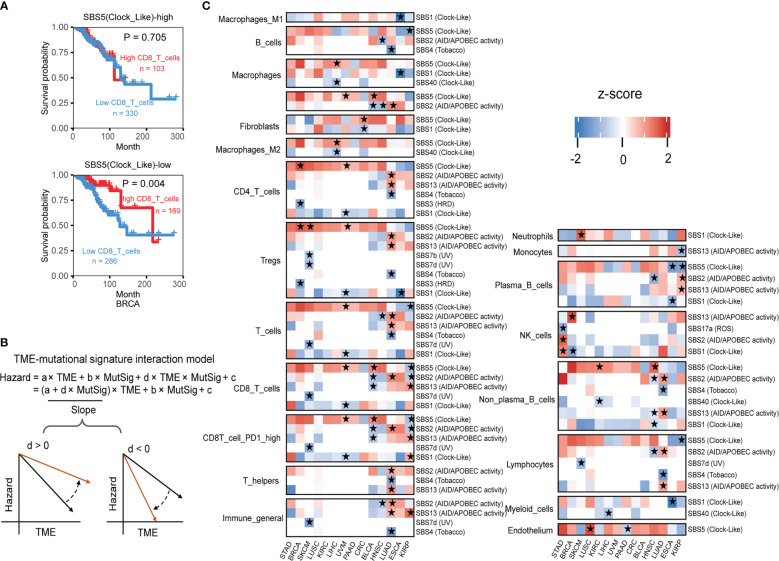
The interaction test identifies mutational signatures of immune phenotype alteration. **(A)** The association between the CD8 T cell level and overall patient survival for kidney renal papillary cell carcinomas with different SBS5 mutational signature activities. Overall survival was analyzed by the two-sided Wald test in the Cox-PH regression to assess the association between TME cell level and overall survival. Samples were categorized by SBS5 mutational signature activity, and were further classified into “High TME cells” (red) and “Low TME cells” (blue) based on TME cell values to show the association between TME cell level and overall survival outcome. **(B)** An interaction test in a Cox-PH regression to identify mutational signatures associated with immune phenotypes. The variable “TME” denotes the level of TME cell infiltration, while “MutSig” represents the activity of a candidate mutational signature. The coefficient “d” reflects the effect of the interaction between TME cells and MutSig on the death hazard outcome estimated from the overall survival data. The graphs depict the association slopes between TME cells and death hazard, with the black and gold arrows indicating the association slopes before and after increasing the level of MutSig. **(C)** Mutational signatures with significant immune phenotype interactions in multiple cancer types. Fourteen cancer types have ten mutational signatures passing the FDR threshold of 0.1. The mutational signatures that have significant immune phenotype interaction scores, defined as the z-score of d/standard error (s.e.), are marked with stars. The two-sided Wald test *P*-values corresponding to an FDR less than 0.1 were used to determine the significance of the mutational signatures. Detailed data are available in **Supplementary Table 9**.

Given few systematic studies jointly exploring mutational signatures and the tumor immune microenvironment, whether their interactions would affect clinical outcomes or not remains largely unclear. We posit that combining tumor mutational signatures, immune status, and patient survival outcomes could reveal key TME regulatory factors. In breast cancer (BRCA), we found that patients with higher CD8 T cell infiltration in the TME have poorer survival outcomes, but only when mutational signature SBS5 activity is low (**Figure 5A**). Wei et al. found that sufficient Clock-like signatures were associated with worse prognosis and the reduction of cytotoxic cell infiltration in melanoma and lung cancer immunotherapy cohorts, increasing the reliability of our results (42).

In statistical terms, the interaction between two variables occurs when one variable’s effect depends on the other variable’s value. In our example, the impact of higher PD1 high CD8 T cell infiltration on survival outcome depends on SBS5 signature activity, illustrating a typical case of variable interaction. By using the Cox interaction model inspired by Jiang et al. study (26), we tested possible interactions between mutational signatures and infiltration of TME cell types on overall survival, with controlling potential confounding factors age, gender, and pathological stage under a false discovery rate (FDR) below 0.1 (**Figure 5B**). Across 14 selected cancer types, ten mutational signatures, including SBS1, SBS2, SBS3, SBS4, SBS5, SBS7b, SBS7d, SBS13, SBS17a and SBS40, showed significant interactions with immune infiltration (**Figure 5C**).

Altogether, our results demonstrate the complex interplay between mutational signatures and immune cell infiltration in the TME and some detail interactions have significant implications for patient survival outcomes, this provides a deeper understanding of mutational signature involved TME regulation and rich resources for further investigations.

### Cancer type-specific interactions between mutational signatures and the tumor microenvironment

**Figure 6:**
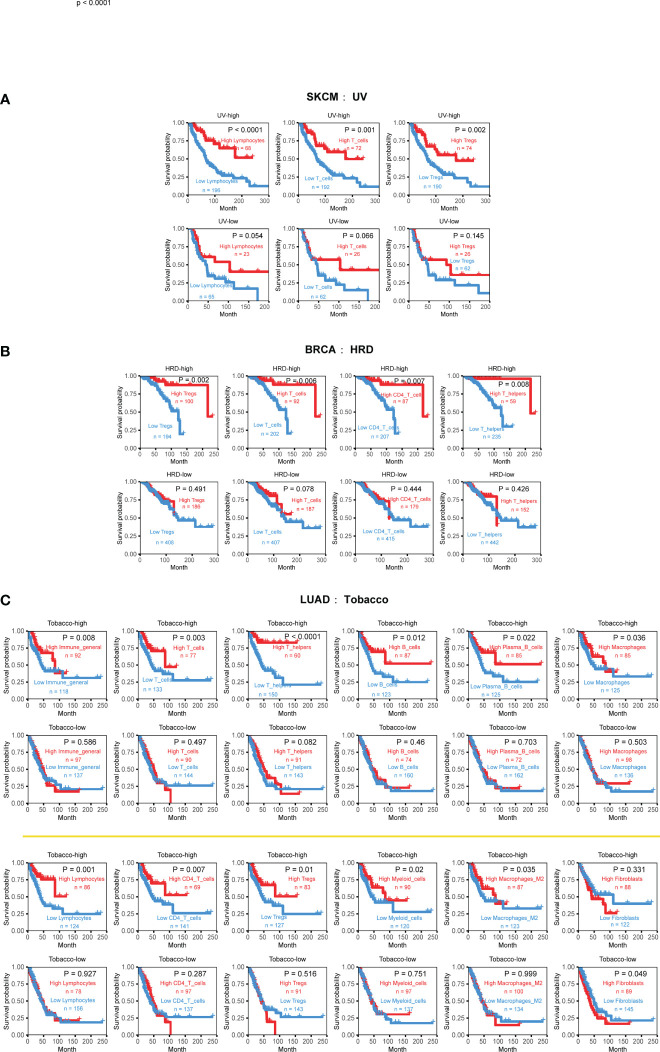
Cancer-type specific mutational signatures are associated with immune phenotypes and patient clinical outcomes. **(A)** Kaplan-Meier curves and log-rank tests were used to evaluate overall survival in 352 skin cutaneous melanoma (SKCM) samples, based on UV-associated mutational signature activity and TME cell infiltration. TME cell levels were classified into high and low abundance groups, using the average infiltration level as the threshold. **(B)** Kaplan-Meier curves and log-rank tests were used to evaluate overall survival in 888 breast cancer (BRCA) samples, according to HRD-associated mutational signature activity and TME cell infiltration. **(C)** Kaplan-Meier curves and log-rank tests were used to evaluate overall survival in 444 lung adenocarcinoma (LUAD) samples, based on tobacco-associated activity and TME cell infiltration. Detailed data are available in **Supplementary Table 9**.

Further analysis based on etiology-associated mutational signatures also shows the widespread impact of mutational signatures on the TME in 11 cancer types (**Supplementary Figure 5A**), such as AID/APOBEC activity, affected three or more cell types of TME in three cancers (**Supplementary Figures 5B, C**). Furthermore, we identified several pathogenic factors that exhibited cancer-specific interaction effects (**Figure 6**).

One example is the strong relationship between UV-associated mutational signature and skin cutaneous melanoma (SKCM). The higher UV exposure, the stronger inhibitory effect of lymphocytes, T cells, and Tregs cells on tumor growth. This observation is consistent with previous reports that UV exposure can affect the genomic and immune phenotypes of SKCM, ultimately promoting cancer occurrence and development (43–45).

Breast cancer (BRCA) is the most common cancer type with HRD phenotype (also named BRCAness). the presence of HRD can affect the occurrence and development of the disease and is closely related to prognosis (46, 47). Comparing to 30 other cancer types, our analysis reveals that the interaction effects of HRD only manifest in breast cancer, typically promoting the beneficial survival effect of T cell, CD4 T cell, Tregs, and Th cell (**Figure 6B**). Patients with high infiltration levels and high HRD signature activity achieved better survival outcomes.

Tobacco-associated mutational signatures in lung cancer had much broader impacts on the TME than the previous two tumors, which is consistent with previous research results (48). Overall, the interaction effect is synergistic, with markedly more substantial immune status and higher exposure will prolong life, especially in myeloid cells, lymphocytes, T cells, B cells, macrophages, and macrophages M2 (**Figure 5A**). Interestingly, fibroblasts display a different form of combined effect in LUAD. When tobacco exposure was low, lower CAF infiltration was associated with a better prognosis (**Figure 6C**). These findings suggest that the impact of mutational signatures on the TME was not uniform across different cancers and might be influenced by cancer-specific factors.

We conducted additional analyses to investigate the influence of mutational signature and tumor microenvironment (TME) interactions on patient survival in three separate cohorts of patients receiving immunotherapy [Van Allen et al., 2015 (49); Hugo et al., 2016 (50); Snyder et al., 2017 (51)]. These include two melanoma datasets and one urethral carcinoma dataset. We observed that UV signatures are both dominant in two melanoma datasets (**Supplementary Figures 6A, B, E, F**) and AID/APOBEC activity is dominant in the urethral carcinoma dataset (**Supplementary Figures 6H, I**). Importantly, in both the Van Allen et al., 2015 (49) and Hugo et al., 2016 (50), the interaction between the TME and the UV signatures affects the patient survival outcome (**Supplementary Figures 6C, D, G**); in Snyder et al., 2017 (51), the interaction between the TME and the APOBEC signatures affects the patient survival outcome (**Supplementary Figures 6J, K**). Significant survival differences were only observed in group with low dominant signature activity. The data underline the impact of mutational signatures on TME constitution and function, emphasizing the potential clinical importance of mutational signatures.

## Discussion

This study investigated the relationship between the well-established SBS mutational signatures and the tumor immune microenvironment across multiple cancer types. Our data revealed that specific mutational signatures could impact the tumor immune microenvironment, and their interaction plays a crucial role in shaping clinical outcomes. Smoking was found to disrupt the immune system’s balance and promote tumor growth and spread, with high levels of tobacco activity associated with a stronger correlation between immune status and better prognosis in LUAD. Homologous recombination deficiency (HRD) was also found to enhance CD8 T cell-mediated killing, which may be important for improving treatment in BRCA and other cancer types. Moreover, a close association between UV radiation and skin cutaneous melanoma (SKCM) was observed. Collectively, this study emphasizes the importance of understanding the interactions of specific mutational signatures and tumor immunophenotypes, and their synergistic or antagonistic effects on patient survival outcomes.

Although previous research has established that high levels of APOBEC mutagenesis are enriched in Bladder (BLCA), head and neck (HNSC), and lung cancers (LUAD and LUSC) (52), the present study demonstrates that the APOBEC signature exerts different interaction effects in these cancers. Specifically, we observed a significant antagonistic effect between APOBEC activity and various types of immune cells in lung adenocarcinoma (LUAD) but not in LUSC, indicating that higher APOBEC levels in tumors could inhibit the beneficial association between high immune infiltration and prolonged overall survival (**Supplementary Figures 5B, C**). Conversely, in head and neck squamous cell carcinoma (HNSC) and bladder urothelial carcinoma (BLAC), there were synergistic effects between APOBEC activity and immune cell infiltration levels in the survival outcomes of cancer patients (**Supplementary Figure 5C**). Patients with high immune cell infiltration levels exhibited favorable prognosis under high APOBEC activity. We also observed a correlation between dMMR-associated mutational signatures and tumor neo-antigen burden, further supporting the formation of new antigens from hypermutation processes and corresponding close changes of immune microenvironments (53–55).

Despite the significance of the findings, the study is not without limitations. First, APOBEC mutagenesis is generally associated with heightened immune activity and improved survival in most cancers, including BLCA (56, 57), HNSC (58), and BRCA (59, 60). However, early evidence suggests that APOBEC correlates with the overexpression of the immune checkpoint molecule PD-L1, which may contribute to the development of immune exhaustion within cancer, leading to poor survival (61). Second, we acknowledge that our sample size, particularly for certain tumor types, may be insufficient for detecting subtle associations between mutational signatures and TME. Third, while our analysis was primarily conducted using TCGA data, we recognize that the lack of other pan-cancer datasets may limit the generalizability of our findings. Fourth, no significantly consistent interactions were observed in the two melanoma immunotherapy datasets. This may due to the insufficient sample size or dataset heterogeneity. Therefore, further research is needed to validate the interaction risk model and address any disputes that may arise regarding the findings. However, despite these limitations, we believe that our study provides valuable insights into the relationship between mutational signatures and TME across multiple cancer types and lays the groundwork for future research in this area.

Although multiple types of mutational signatures including SBS signatures, double-base substitution (DBS) signatures, insertion-deletion (INDEL) signatures and copy number signatures have been proposed (1, 62), only well-established SBS signatures are considered in this study due to several reasons. Firstly, the sample size of TCGA with available information on DBS signatures and INDEL signatures is very low compared to SBS signatures. This is because DBS and INDEL mutations are much less frequent than SBS mutations, which limits our ability to obtain reliable results across different tumor types and pan-cancer analyses. Therefore, we excluded these signatures from our initial explorations. Secondly, we did explore copy number variation (CNV) signatures, but we did not observe any positive results, particularly regarding the interaction between CNV patterns and the composition of immune microenvironments. As a result, we did not include CNV signatures in our presented data. Expanding our analysis to include other mutational signatures in future studies if sample sizes allow could potentially provide further insights into the relationship between mutational processes and the immune system.

In conclusion, this study contributes significantly to understanding the complex interaction between mutational signatures and the tumor immune microenvironment, which can potentially improve precision cancer therapy. Further research is warranted to validate these findings and devise novel strategies that can specifically target mutational signatures and immune cell components for improved clinical outcomes. Additionally, more extensive investigations should explore the molecular mechanisms underlying the interaction between mutational signatures and the tumor immune microenvironment to identify promising targets for the development of novel therapies, ultimately leading to improved cancer treatment efficacy and patient outcomes.

## Methods

### Data collection

In this study, we analyzed data of cancer patients from The Cancer Genome Atlas (TCGA) project. Patient clinical information including survival data were acquired from R package UCSCXenaShiny (63). Mutational signature activities were obtained from Alexandrov et al. (2). Kassandra decomposed tumor microenvironment estimations were obtained from (32), EPIC (64), and quanTIseq (65) decomposed tumor microenvironment estimations were obtained from TIMER 2.0 database (http://timer.cistrome.org) (38). Three immunotherapy genomics datasets including Van Allen et al., 2015 (49), Hugo et al., 2016 (50), and Snyder et al., 2017 (51) were collected from the TIGS study (66). In more detail, the Van Allen et al. (2015) (49) study is a melanoma immune cohort targeting CTLA-4, including 110 whole-exome sequencing samples, 110 patient clinical information, and 43 RNA-seq samples. The Hugo et al. (2016) (50) study targets PD-1 in the melanoma immune cohort, including 38 whole-exome sequencing samples, 37 patient clinical information, and 27 RNA-seq samples. Lastly, the Snyder et al. (2017) (51) study is a urothelial cancer cohort targeting PD-L1, including 25 whole-exome sequencing samples, 25 patient clinical information, and 25 RNA-seq samples.

### Data preprocessing

TCGA cohort LAML (Acute Myeloid Leukemia) was excluded from our analysis due to the distinctive immunological status. COAD and READ were combined into CRC. In total, 31 cancer types were included in our study. Cancer patients with data of both SBS mutational signature activities and Kassandra decomposed tumor microenvironment estimations were kept. Next, we removed SBS mutational signatures which are categorized as “Possible sequencing artefacts” in COSMIC mutational signature database (https://cancer.sanger.ac.uk/signatures/sbs/). Furthermore, we only kept SBS mutational signatures with non-zero signature activity exceeded 15% of the total sample size for each cancer type. The mutational signature activities were normalized by Z-score method and scaled to range 0 to 1. The activities of etiology-associated mutational signatures were calculated as the sum of signature activities of SBS mutational signatures with the same etiology (**Supplementary Table 1**), such as concurrent POLE/POLD1 mutations, concurrent polymerase epsilon mutation, DNA mismatch repair and MSI were attributed to dMMR-associated mutational signatures. We excluded mutational signatures with “unknown” etiology for subsequent analysis to enhance the data interpretability. For our analysis, we used immune infiltration data from tumor samples that matched the filtered mutation data mentioned above. To ensure the reliability, accuracy, and biological significance of our results, we primarily use TME data from Aleksandr et al. (2022) (32). We retained 21 cell types in the Kassandra algorithm: B cells, CD4 T cells, CD8 T cells, CD8 T cells PD1 high, CD8 T cells PD1 low, Endothelium, Fibroblasts, Immune general, Lymphocytes, Macrophages, Macrophages M1, Myeloid cells, NK cells, Neutrophils, Non-plasma B cells, T helpers, and Tregs. We also included seven cell types identified by the EPIC method: B cells, CAFs, CD4+ T cells, CD8+ T cells, Endothelium, Macrophages, and NK cells; and four cell types detected by the quanTIseq method: B cells, NK cells, CD8+ T cells, and Tregs.

### Association analysis between mutational signatures and the tumor microenvironment estimations

A linear regression model was constructed for each TME cell type by using the mutational signature matrix and a matrix containing one-hot encoded patient-specific cancer type as covariable. To test if clinical factors could significantly impact the regression results, we also built a similar model, but with age, gender and tumor stage included as covariates. We performed individual corrections for each type of cancer to get the values of false discovery rate (FDR). The result data were visualized by R package ComplexHeatmap (67).

### Least absolute shrinkage and selection operator analysis

*Prediction of tumor immune infiltration*. Firstly, we selected immune-related mutational signatures, defined as those with FDR-corrected *P*-values < 0.1 from previous association analysis. Subsequently, LASSO linear regression was performed using R package glmnet (v4.1.4) (68), with 10-fold cross-validation to select the most relevant subset of features for predicting immune cell infiltration across cancer types. The optimal penalty parameter was chosen based on the lambda value corresponding to the minimum mean cross-validation error. In the event that none of the signatures were significant after selection, this may indicate high collinearity, and all variables were retained as the optimal features. Linear regression was used to obtain the TME prediction value when only one variable was included in the LASSO selection process. To facilitate the use of this approach, we developed a user-friendly R language-based prediction function available at https://github.com/luolz/TME-mutational-signature-interaction. Tumors were also grouped based on their average TME cell type prediction values, and we used Wilcoxon tests to examine differences in the true infiltration values between the two groups in different cancer types.

*Constructing risk scores*. We determined the immune cells significantly associated with overall survival (OS) and then extracted all related mutational signatures in each cancer type for subsequent analysis. For each type of cancer, we conducted LASSO Cox regression with 10-fold cross-validation to select prognostic mutational signatures. The predictors with non-zero coefficient were used to compute the risk score in the final model. If none of the predictors are significant after selection, all variables were retained. The risk score based on etiology mutational signatures was calculated in the same manner.

### Modeling the survival hazard associated interaction of mutational signatures and cell types in the tumor microenvironment

In this analysis, we included cancer types with more than 50 patients and a mortality rate greater than 10%. Furthermore, we kept cancer types that both SBS-high and SBS-low groups have at least 20 samples. In total, 18 cancer types were selected, including ACC, BLCA, BRCA, CHOL, CRC, ESCA, HNSC, KICH, KIRC, KIRP, LIHC, LUAD, LUSC, MESO, PAAD, SKCM, STAD and UVM. We then used multivariate Cox-PH regression and applied an interaction test to assess if mutational signatures interact with TME cell types to affect survival outcomes. Potential confounding factors were incorporated into modeling covariates, such as age, gender, and pathological stage. We defined the TME-MutSig interaction score for each mutational signature using the Wald test z-score, which is the coefficient d divided by its standard error. We used the Benjamini-Hochberg method to convert two-sided Wald test *P*-values to FDRs and identified significant mutational signatures in cancer types with over 1% mutational signatures and FDR below 0.1. To confirm the existence of interaction effect between mutational signatures and the tumor microenvironment, we performed survival analysis on the significant mutational signatures (*P*<0.05) to compare the difference of distinct groups with a log-rank test.

### Exploration of interaction effects in immunotherapy cohort data

We obtained the original mutation data for the immunotherapy cohort from the relevant literature and acquired the MAF file after ANNOVAR (69) annotation. We used the Sigminer package (70) to obtain 72 SBS signatures corresponding to the COSMIC V3 version (1). Following the filtering process, we retained 47 SBS for further analysis. We acquired the normalized gene-level RNA-Seq data and clinical information following the steps outlined by Wang et al (66). Since the Kassandra method is not applicable for the immunotherapy datasets, here we computed the EPIC immune infiltration according to the workflow of the IOBR package (71).

For final processed data, in the Van Allen et al. (2015) (49), Hugo et al. (2016) (50), and Snyder et al. (2017) (51) cohorts, the number of samples with mutational signatures, immune infiltration, and survival information are 40 samples, 26 samples, and 22 samples, respectively. Due to the lack of data in the immunotherapy cohort and the absence of clinical information for some samples, we did not include age, gender, and stage as covariates when testing the interaction effect. The remaining steps were carried out as before.

### Statistical analysis

Correlation analysis was performed using the Spearman correlation coefficient. Kaplan-Meier survival curves were depicted to compare the difference of distinct groups with a log-rank test. All reported *P*-values are two-tailed, *P*<0.05 is considered statistically significant, unless otherwise specified. Multiple testing *P*-values were corrected by Benjamini–Hochberg FDR method, with FDR<0.1 as the significant threshold. All statistical analyses of this study were implemented in R v4.0.1.

## Data availability statement

The original contributions presented in the study are included in the article/Supplementary Material. All original data are available at Zenodo (https://doi.org/10.5281/zenodo.7912835). The analysis code has been deposited in GitHub at https://github.com/luolz/TME-mutational-signature-interaction. For any further inquiries, please contact the corresponding author.

## Ethics statement

Ethical review and approval was not required for the study on human participants in accordance with the local legislation and institutional requirements. The patients/participants provided their written informed consent to participate in this study.

## Author contributions

Study concept and design: SW and QZ. Acquisition of data: SW, SL, CW, JM, LQ, and YC. Analysis and interpretation of data: ZL. Drafting of the manuscript: ZL, SW, CW, JM, LQ, and QZ. Critical revision of the manuscript for important intellectual content: ZL, SL, SW, and QZ. Obtaining funding: SW and QZ. Technical or material support: SW and QZ. Supervision: SW and QZ. All authors contributed to the article and approved the submitted version.

## Supplementary material

Supplementary Figure 1 Picture and validation of interplay between mutational signatures and the immune microenvironment in different cancer types. **(A-C)** Heatmaps showing association p values between SBS mutational signatures and TME cell types derived from approaches **(A)** Kassandra, **(B)** EPIC, and **(C)** quanTIseq. **(D-F)** Heatmaps showing association p values between etiology-associated mutational signatures and TME cell types derived from approaches **(D)** Kassandra, **(E)** EPIC, and **(F)** quanTIseq. Detailed data are available in **Supplementary Tables 3**, **5**, **6**.

Supplementary Figure 2 Validation of the relationship between mutational signatures and cell types of TME. **(A)** Association map between SBS mutational signatures and cell types in tumor microenvironment decomposed by Kassandra, using age, gender, and tumor stage as covariates. The threshold is FDR < 0.1. **(B)** Association map between etiology-associated mutational signatures and cell types in tumor microenvironment decomposed by Kassandra, using age, gender, and tumor stage as covariates. The threshold is FDR < 0.1. **(C)** Association map between SBS mutational signatures and cell types in tumor microenvironment decomposed by EPIC. The threshold is FDR < 0.1. **(D)** Association map between etiology-associated mutational signatures and cell types in tumor microenvironment decomposed by EPIC. The threshold is FDR < 0.1. **(E)** Association map between SBS mutational signatures and cell types in tumor microenvironment decomposed by quanTIseq. The threshold is FDR < 0.1. **(F)** Association map between etiology-associated mutational signatures and cell types in tumor microenvironment decomposed by quanTIseq. The threshold is FDR < 0.1. **(G)** Color coding for cancer types.

Supplementary Figure 3 Validation of predicting tumor immune infiltration with LASSO regression modeling on mutational signatures. **(A)** Predicting EPIC decomposed immune cell infiltration of the samples. The darker the color, the higher the correlation between the predicted value and the real value. **(B)** Boxplot estimating the stratification ability of EPIC decomposed immune cell infiltration with predicted immune cell infiltration status. **(C)** Predicting quanTIseq decomposed immune cell infiltration of the samples. The darker the color, the higher the correlation between the predicted value and the real value. **(D)** Boxplot estimating the stratification ability of quanTIseq decomposed immune cell infiltration with predicted immune cell infiltration status. Detailed data are available in **Supplementary Table 7**.

Supplementary Figure 4 Stratification of patient survival based on etiology-associated mutational signatures. **(A)** quanTIseq and **(B)** EPIC decomposed TME cell types associated with pan-cancer survival. **(C, D)** Kaplan–Meier curve showing survival associated with risk score in cancer types **(C)** CESC and **(D)** UCS. **(E)** Forest plot displaying the associations between immune-related and etiology-associated mutational signatures derived risk scores and patient overall survival using univariate Cox regression analysis. **(F)** Kaplan–Meier curve showing survival associated with immune-related and etiology-associated mutational signatures derived risk score among 17 cancer types. Detailed data are available in **Supplementary Table 8**.

Supplementary Figure 5 Etiology-associated mutational signatures of immune phenotype alteration. **(A)** Etiology-associated mutational signatures with significant immune phenotype interactions in multiple cancer types. **(B, C)** Kaplan-Meier curves and log-rank tests were respectively used to evaluate overall survival in 444 LUAD samples, 397 HNSC samples and 380 HNSC samples, based on AID/APOBEC-associated mutational signature activity and TME cell infiltration. Detailed data are available in **Supplementary Table 9**.

Supplementary Figure 6 Specific mutational signatures are associated with immune phenotype alteration in immunotherapy cohorts. **(A)** Box plot of SBS mutational signatures of samples in the Van Allen study. **(B)** Box plot of etiology-associated mutational signatures of samples in the Van Allen study. **(C, D)** Kaplan-Meier curves and log-rank tests were used to evaluate overall survival in 40 melanoma immunotherapy samples based on SBS7b, UV-associated mutational signature activity, and TME cell infiltration. **(E)** Box plot of SBS mutational signatures of samples in the Hugo study. **(F)** Box plot of etiology-associated mutational signatures of samples in the Hugo study. **(G)** Kaplan-Meier curves and log-rank tests were used to evaluate overall survival in 26 melanoma immunotherapy samples based on SBS7b, SBS7c, and TME cell infiltration. **(H)** Box plot of SBS mutational signatures of samples in the Snyder study. **(I)** Box plot of etiology-associated mutational signatures of samples in the Snyder study. **(J, K)** Kaplan-Meier curves and log-rank tests were used to evaluate overall survival in 22 urothelial immunotherapy samples based on SBS13, AID/APOBEC-associated mutational signature activity, and TME cell infiltration. Detailed data are available in **Supplementary Table 10**.

Supplementary Table 1 Classification of SBS mutational signatures according to their etiologies.

Supplementary Table 2
*P*-value scaling guidelines for significance representation.

Supplementary Table 3 Regression model results of original, scaled, and FDR-adjusted *P*-value with Kassandra-derived TME dataset.

Supplementary Table 4 The number of cell associations.

Supplementary Table 5 Regression model results of original, scaled, and FDR-adjusted *P*-value with EPIC-derived TME dataset.

Supplementary Table 6 Regression model results of original, scaled, and FDR-adjusted *P*-value with quanTIseq-derived TME dataset.

Supplementary Table 7 Results of correlation and Wilcoxon test for TME prediction model.

Supplementary Table 8 Results of risk score and survival analysis.

Supplementary Table 9 Results of interaction effects and associated survival analysis in TCGA cohort.

Supplementary Table 10 Results of interaction effects and associated survival analysis in immunotherapy cohorts.
