# Supplementary figures and images for: Unveiling the interplay between mutational signatures and tumor microenvironment: a pan-cancer analysis
Source: Front Immunol. 2023 May 22;14:1186357. doi: 10.3389/fimmu.2023.1186357 (PMC10239828; doi:10.3389/fimmu.2023.1186357)

A

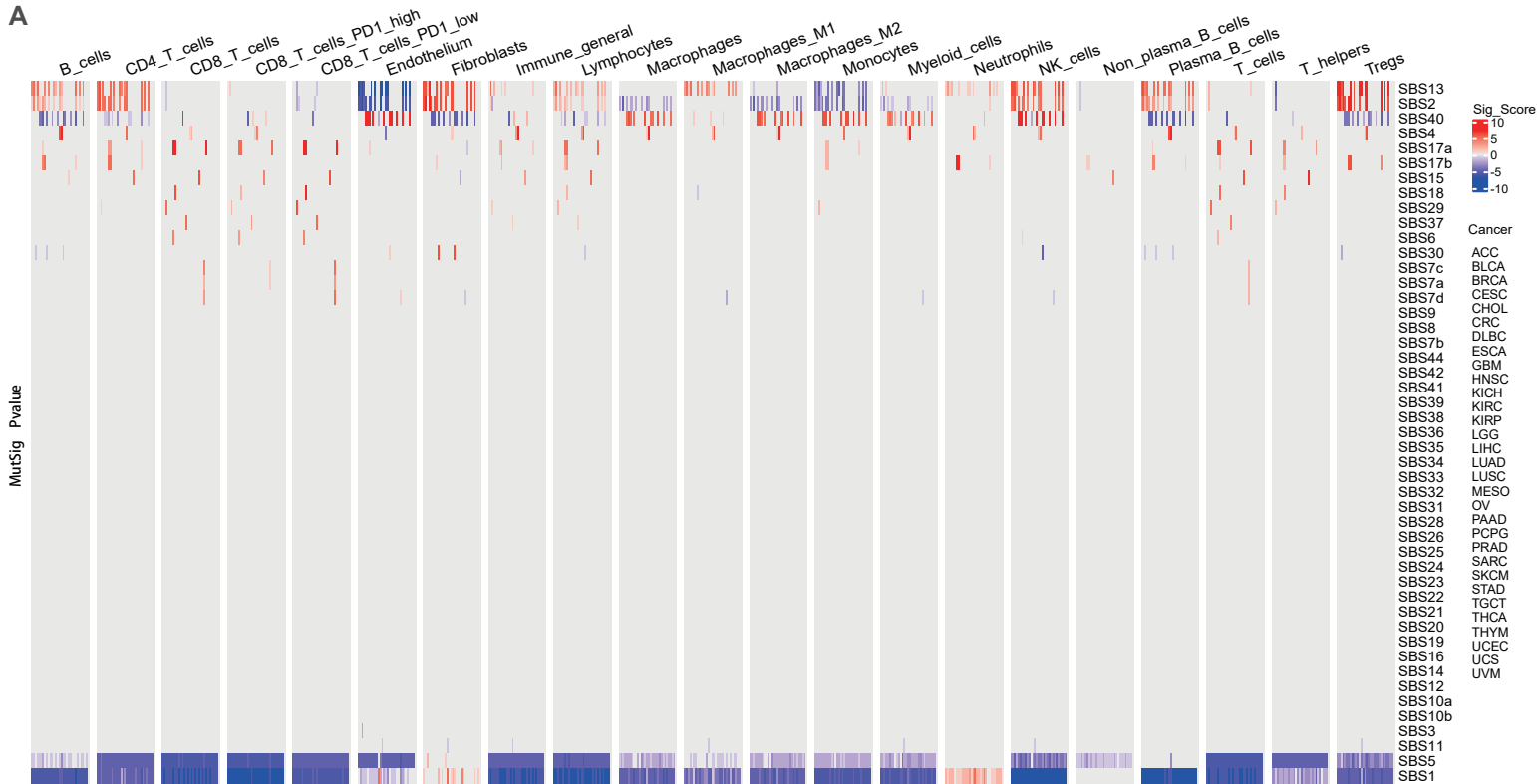

B

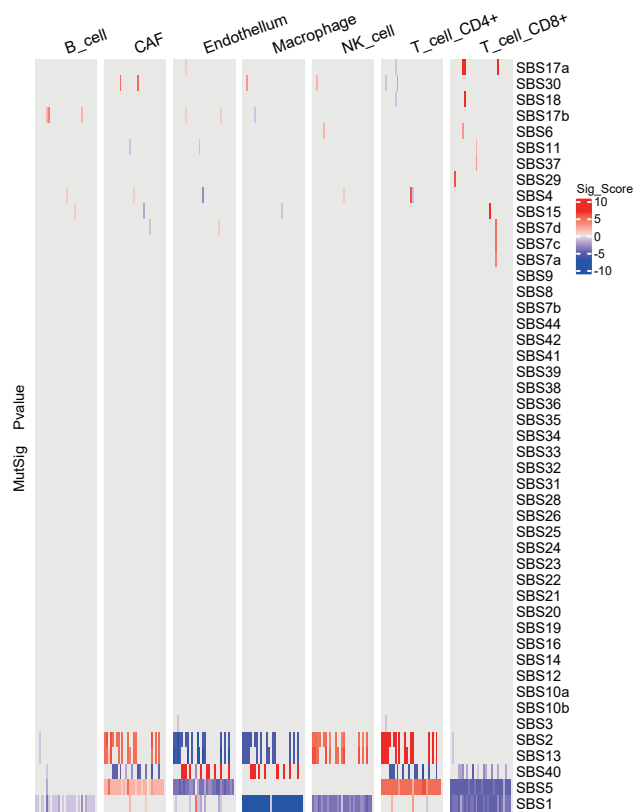

C

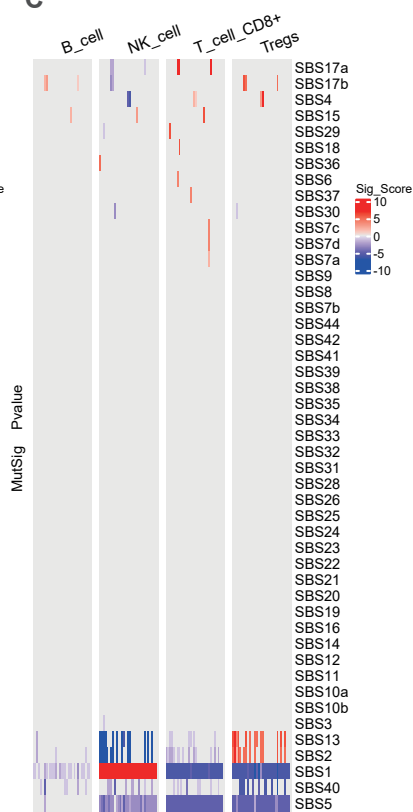

E

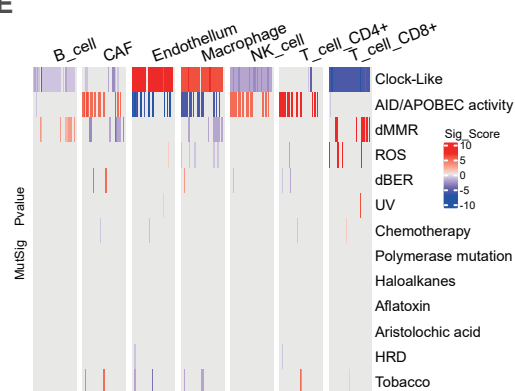

F

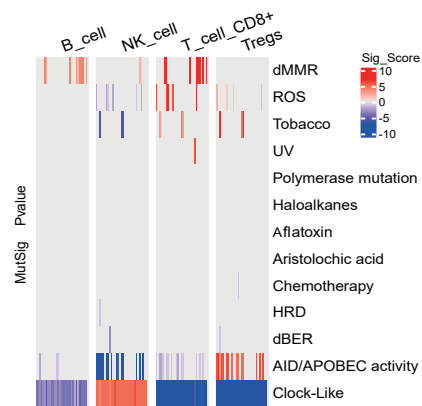

D

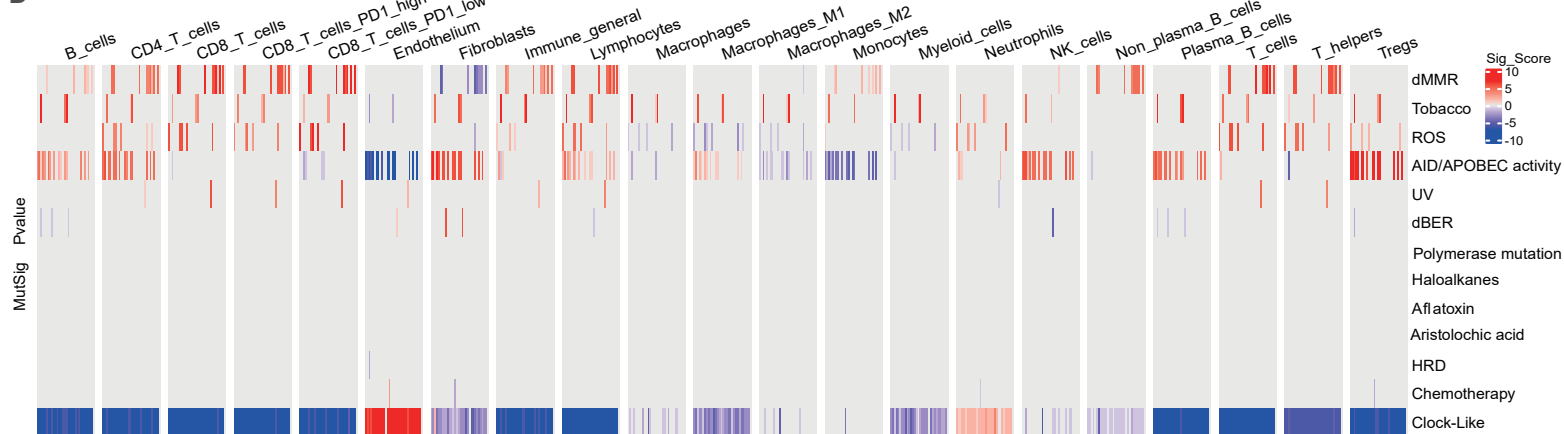

Supplement: Supplementary Figure 1 — Picture and validation of interplay between mutational signatures and the immune microenvironment in different cancer types. (A-C) Heatmaps showing association p values between SBS mutational signatures and TME cell types derived from approaches (A) Kassandra, (B) EPIC, and (C) quanTIseq. (D-F) Heatmaps showing association p values between etiology-associated mutational signatures and TME cell types derived from approaches (D) Kassandra, (E) EPIC, and (F) quanTIseq. Detailed data are available in Supplementary Tables 3 , 5 , 6 . [file DataSheet_1.zip › Supplementary_files/Supplementary_Fig1.pdf]

# A

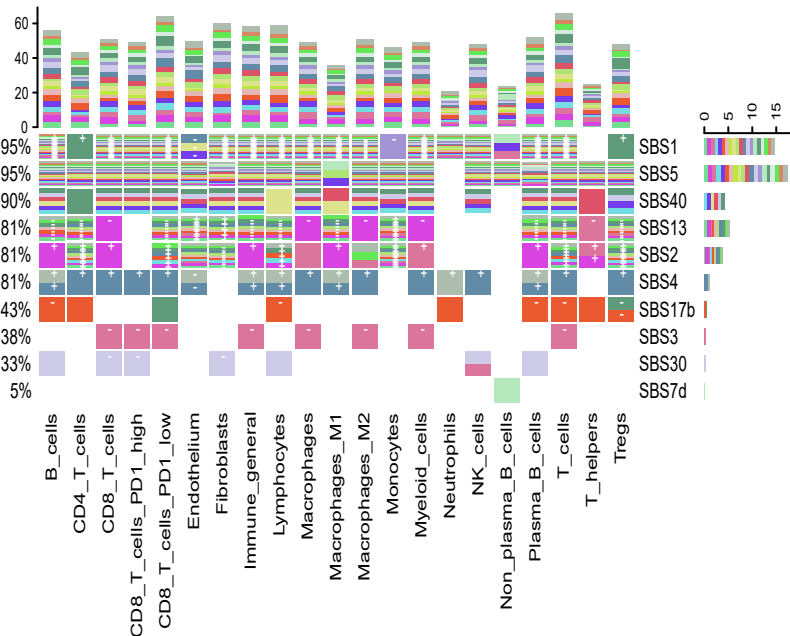

B

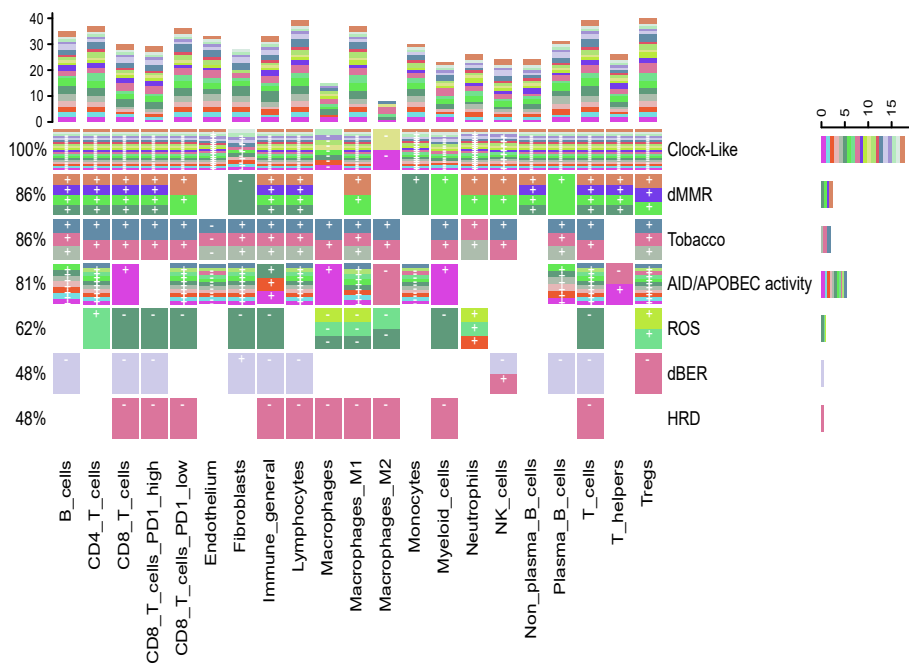

## E

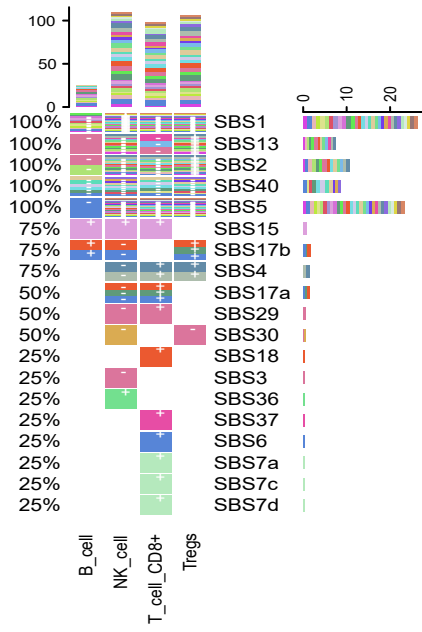**F**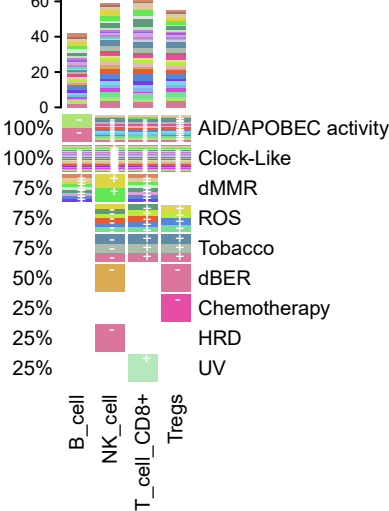

C

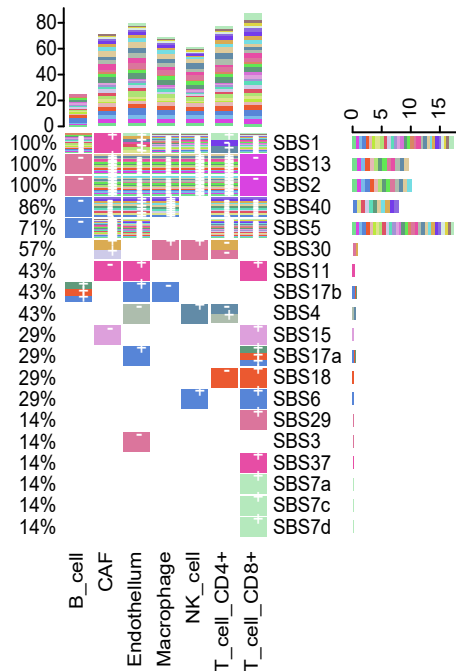

D

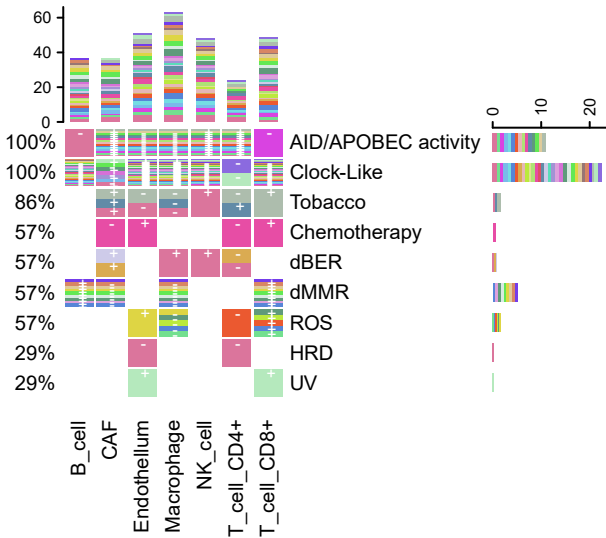

**G**

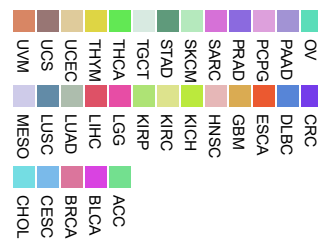

Supplement: Supplementary Figure 1 — Picture and validation of interplay between mutational signatures and the immune microenvironment in different cancer types. (A-C) Heatmaps showing association p values between SBS mutational signatures and TME cell types derived from approaches (A) Kassandra, (B) EPIC, and (C) quanTIseq. (D-F) Heatmaps showing association p values between etiology-associated mutational signatures and TME cell types derived from approaches (D) Kassandra, (E) EPIC, and (F) quanTIseq. Detailed data are available in Supplementary Tables 3 , 5 , 6 . [file DataSheet_1.zip › Supplementary_files/Supplementary_Fig2.pdf]

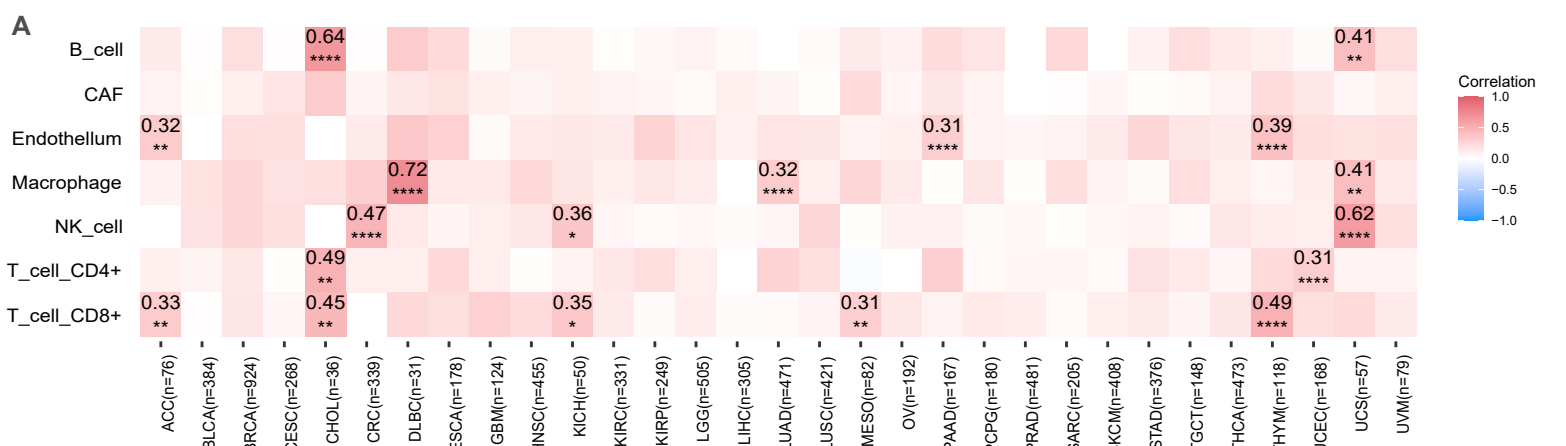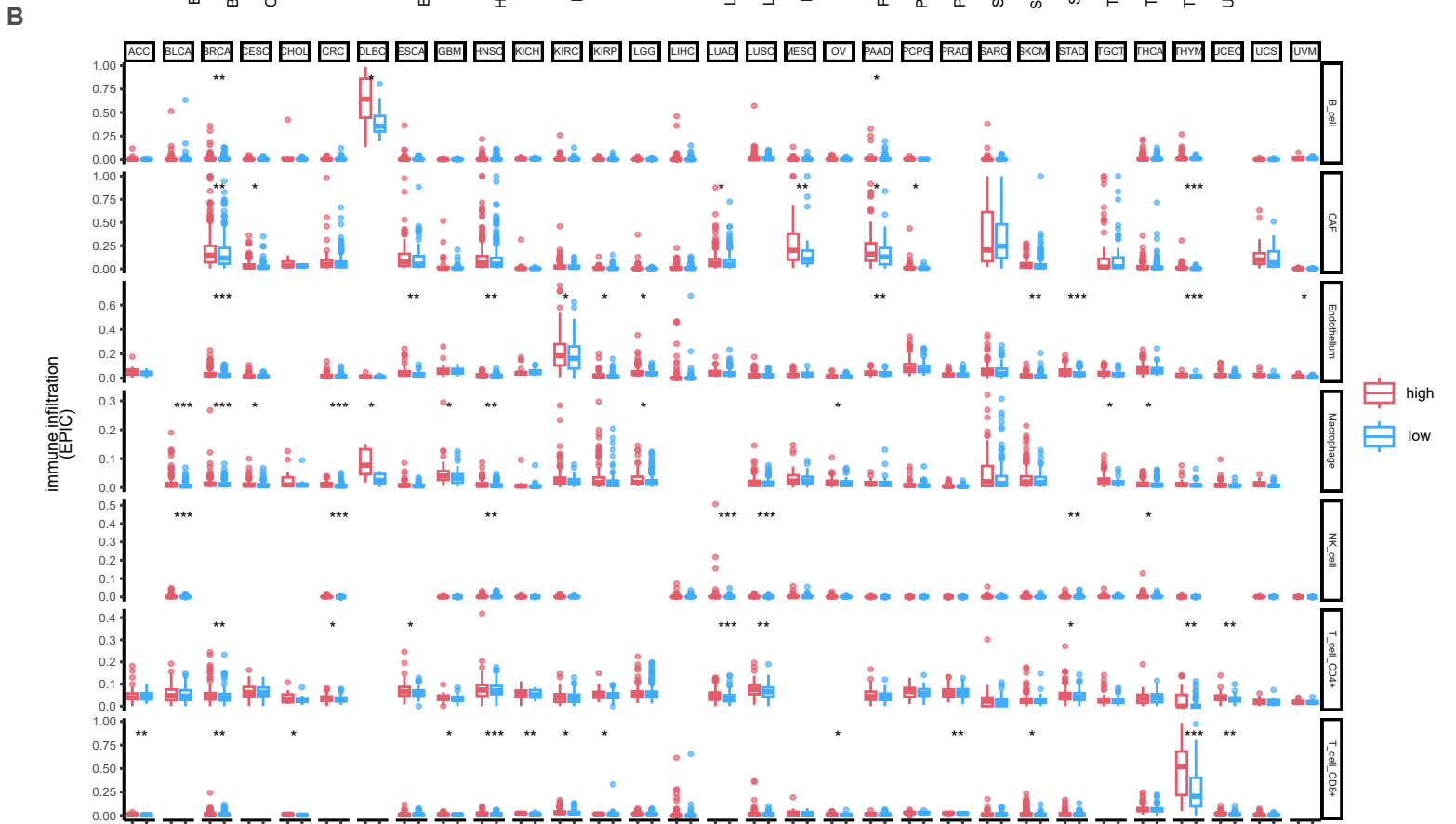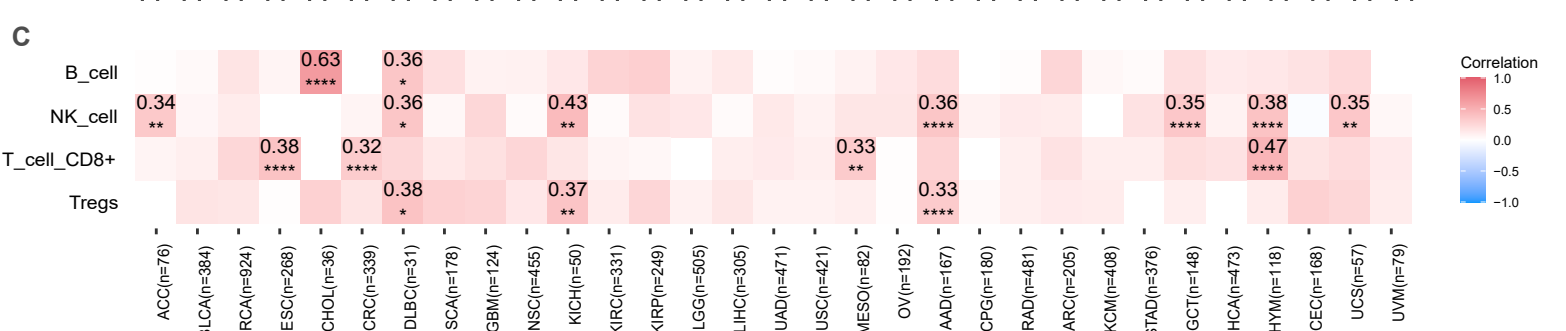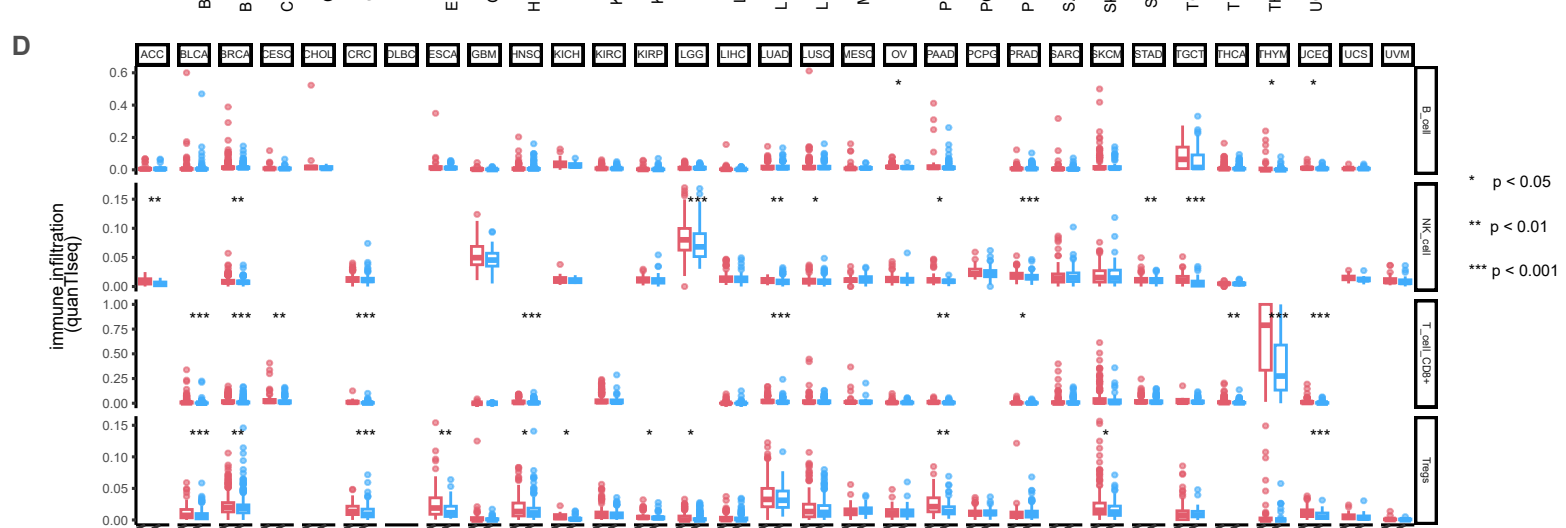

Supplement: Supplementary Figure 1 — Picture and validation of interplay between mutational signatures and the immune microenvironment in different cancer types. (A-C) Heatmaps showing association p values between SBS mutational signatures and TME cell types derived from approaches (A) Kassandra, (B) EPIC, and (C) quanTIseq. (D-F) Heatmaps showing association p values between etiology-associated mutational signatures and TME cell types derived from approaches (D) Kassandra, (E) EPIC, and (F) quanTIseq. Detailed data are available in Supplementary Tables 3 , 5 , 6 . [file DataSheet_1.zip › Supplementary_files/Supplementary_Fig3.pdf]

A

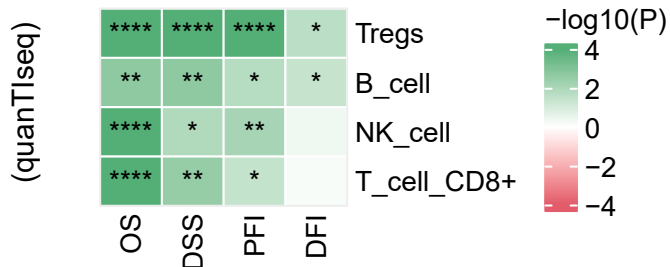

B

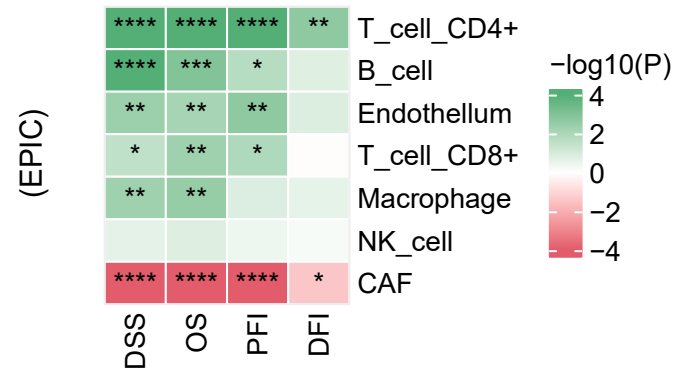

C

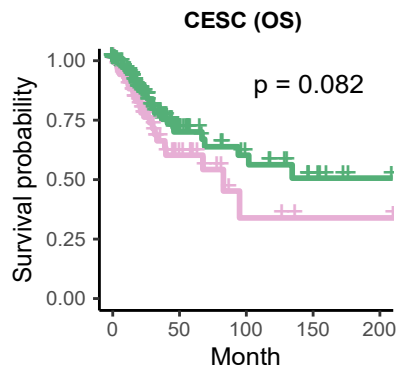

D

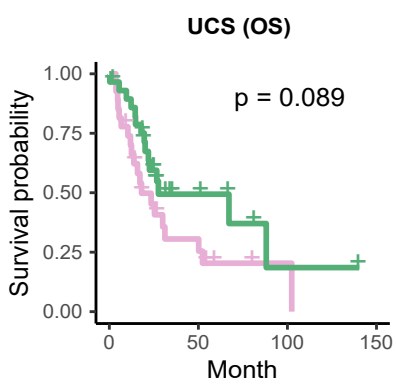

E

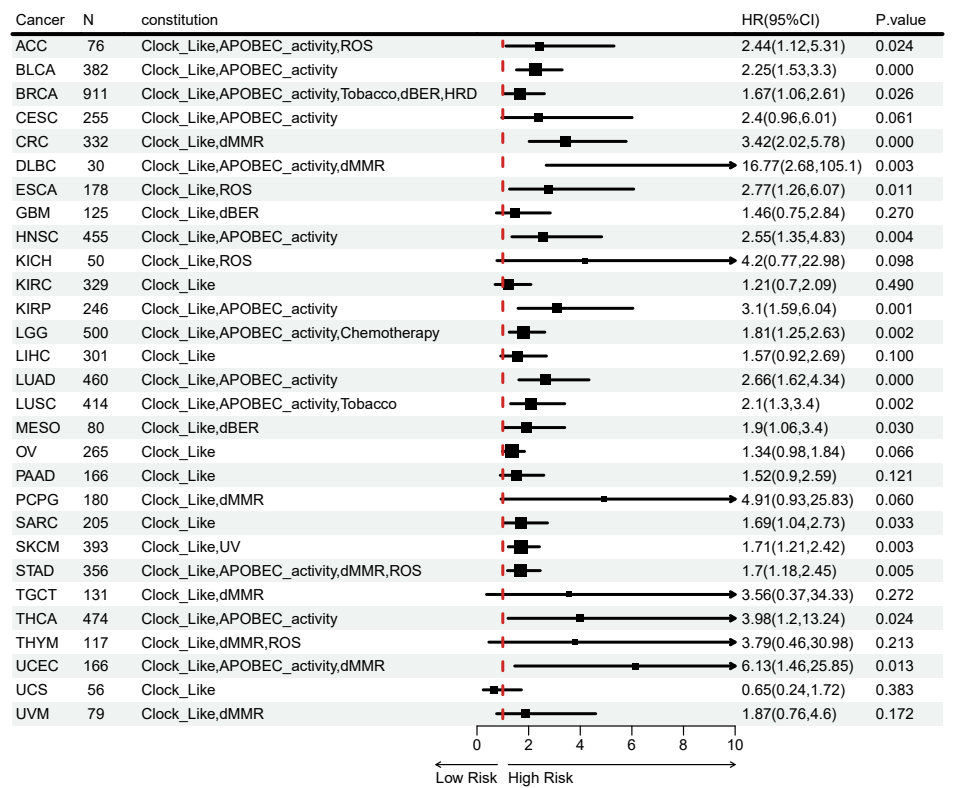

F

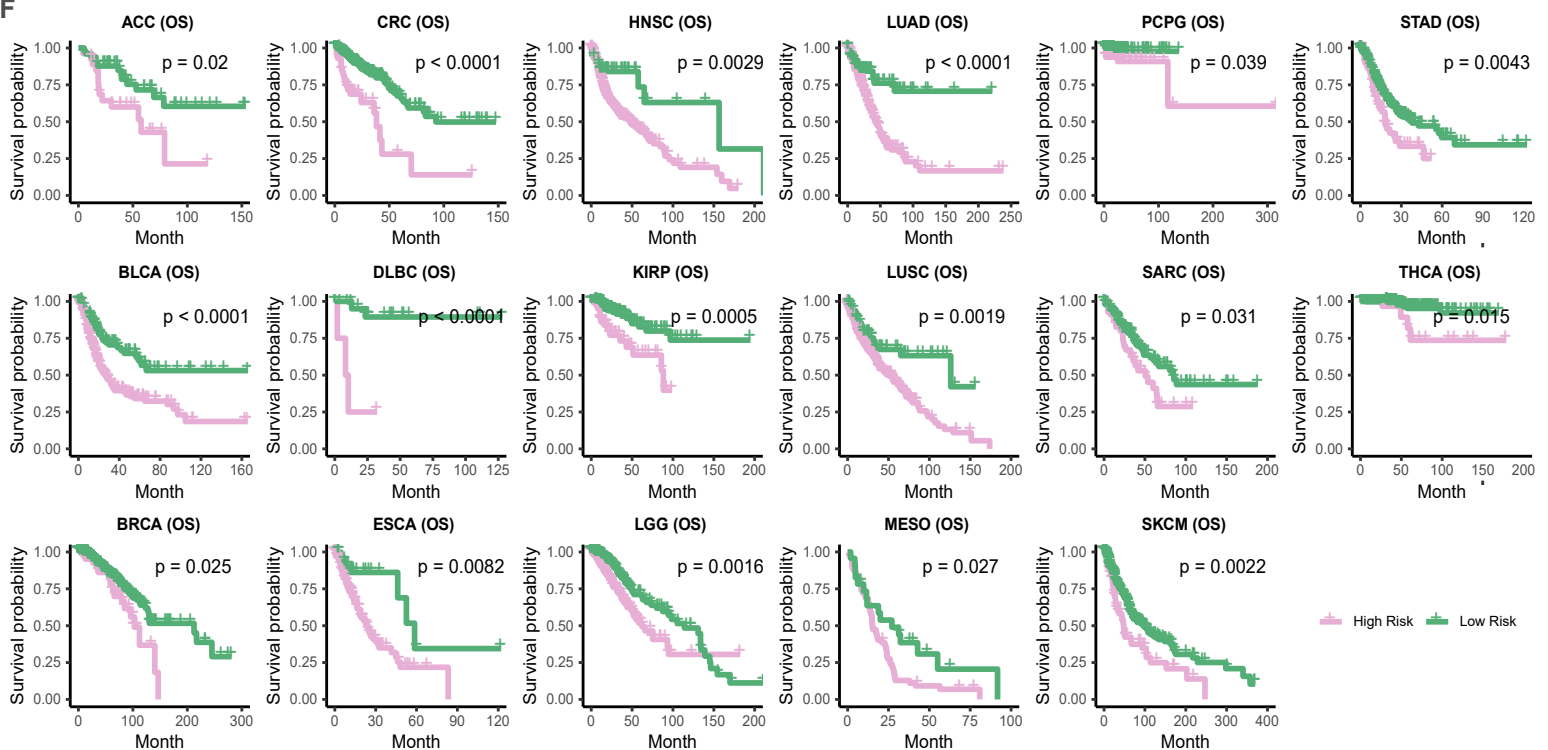

Supplement: Supplementary Figure 1 — Picture and validation of interplay between mutational signatures and the immune microenvironment in different cancer types. (A-C) Heatmaps showing association p values between SBS mutational signatures and TME cell types derived from approaches (A) Kassandra, (B) EPIC, and (C) quanTIseq. (D-F) Heatmaps showing association p values between etiology-associated mutational signatures and TME cell types derived from approaches (D) Kassandra, (E) EPIC, and (F) quanTIseq. Detailed data are available in Supplementary Tables 3 , 5 , 6 . [file DataSheet_1.zip › Supplementary_files/Supplementary_Fig4.pdf]

**A**

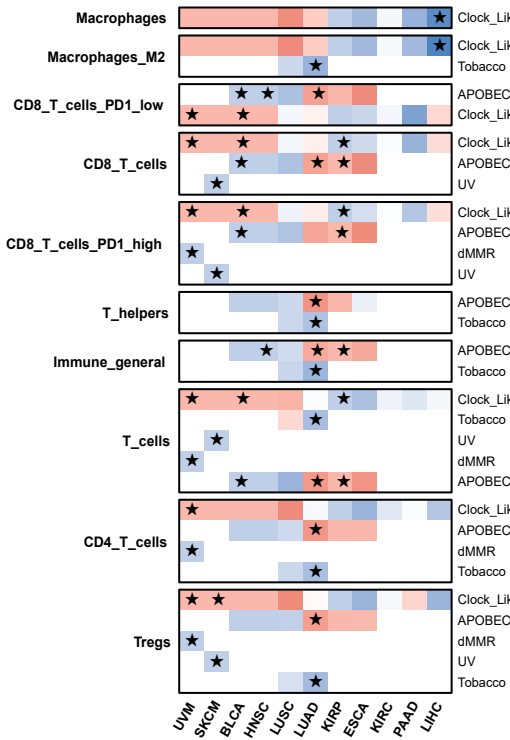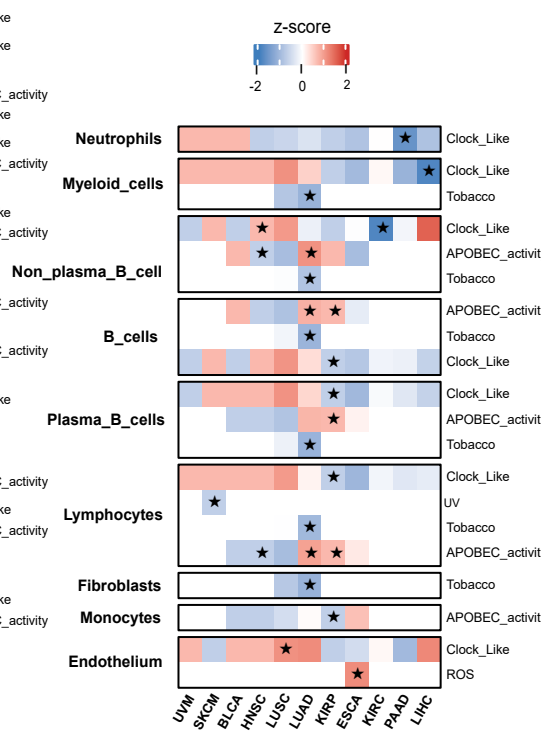

**B**

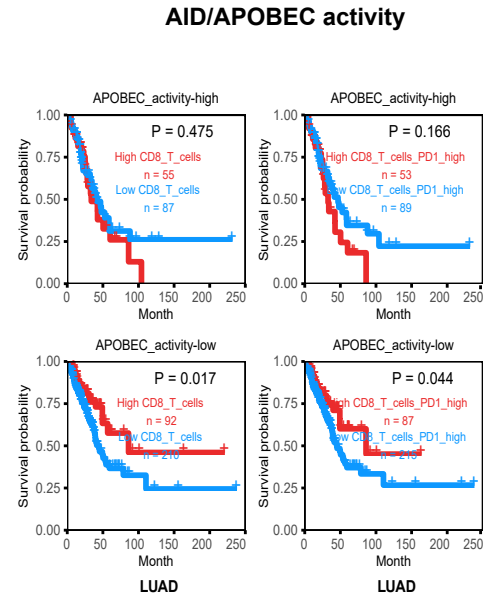

**C**

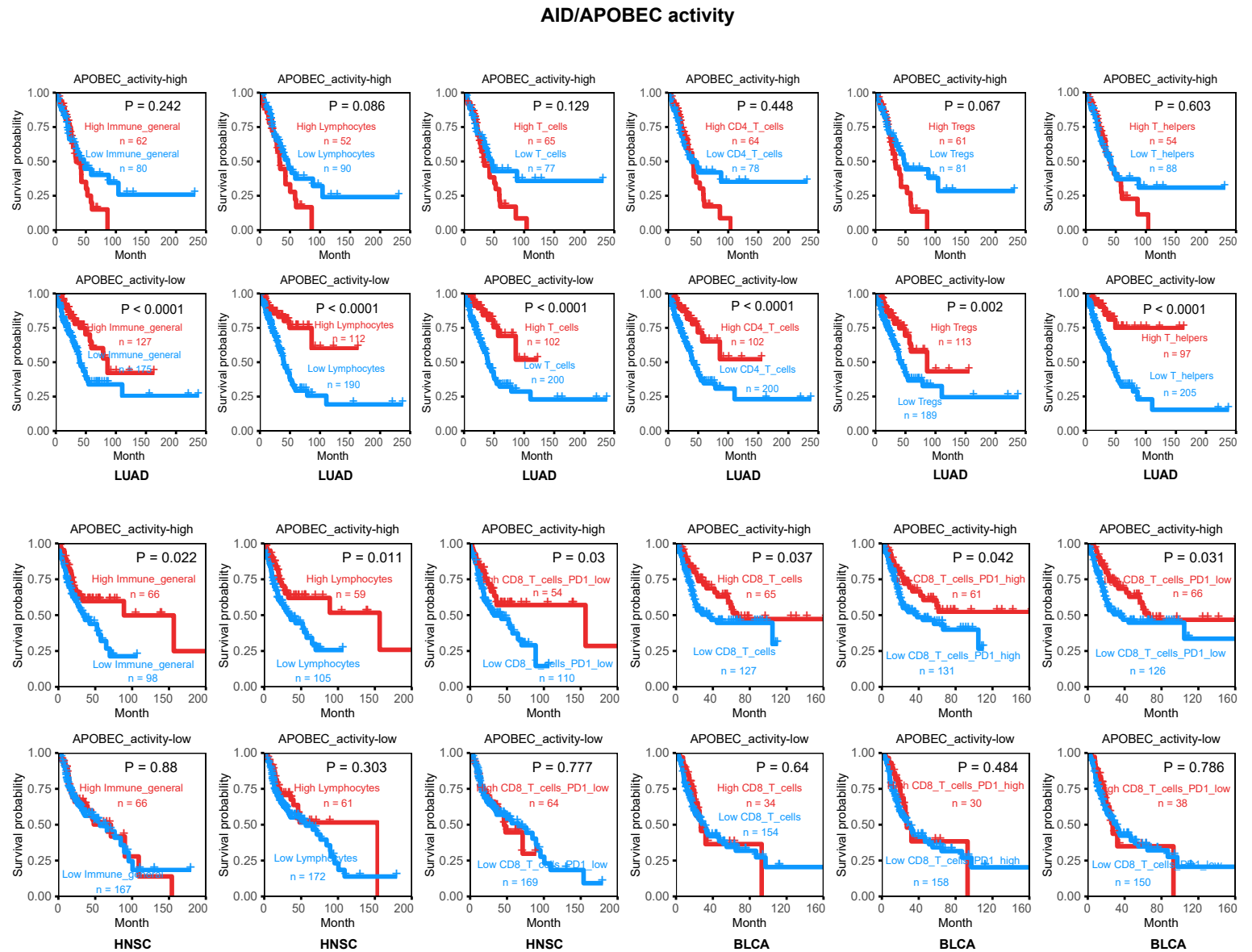

Supplement: Supplementary Figure 1 — Picture and validation of interplay between mutational signatures and the immune microenvironment in different cancer types. (A-C) Heatmaps showing association p values between SBS mutational signatures and TME cell types derived from approaches (A) Kassandra, (B) EPIC, and (C) quanTIseq. (D-F) Heatmaps showing association p values between etiology-associated mutational signatures and TME cell types derived from approaches (D) Kassandra, (E) EPIC, and (F) quanTIseq. Detailed data are available in Supplementary Tables 3 , 5 , 6 . [file DataSheet_1.zip › Supplementary_files/Supplementary_Fig5.pdf]

**A**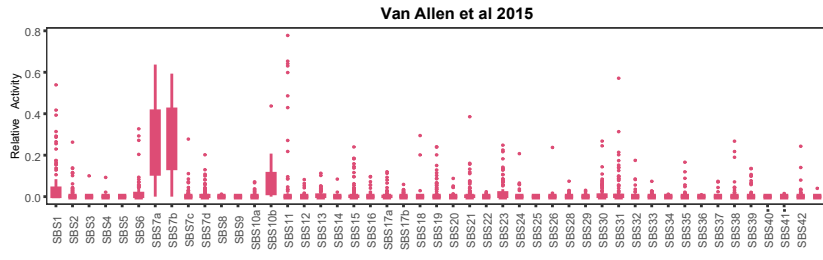**B**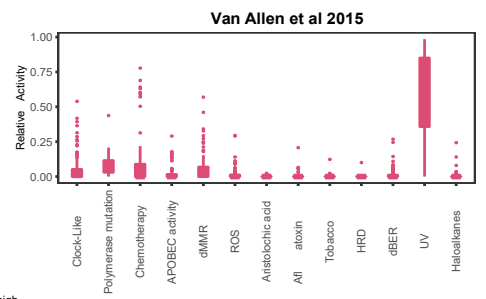**C**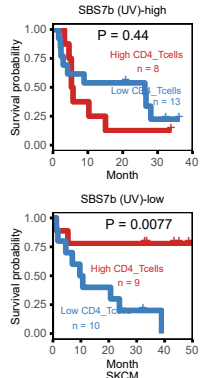**D**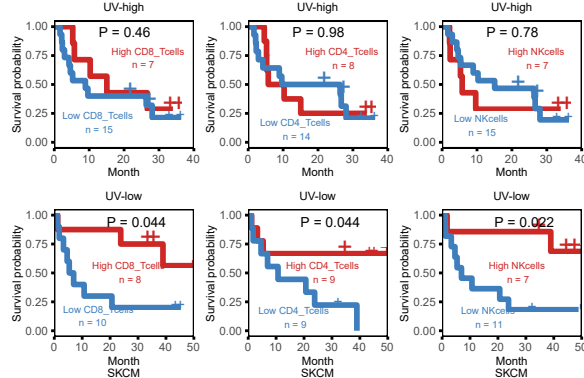**E**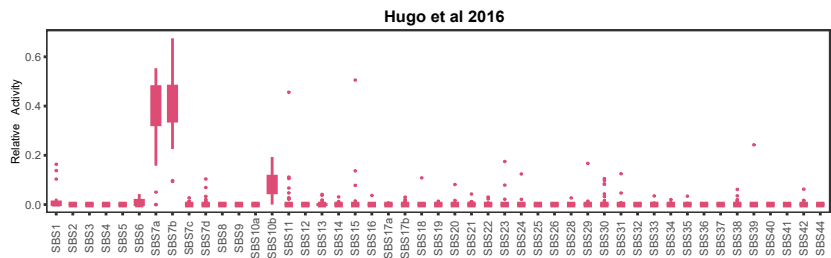**F**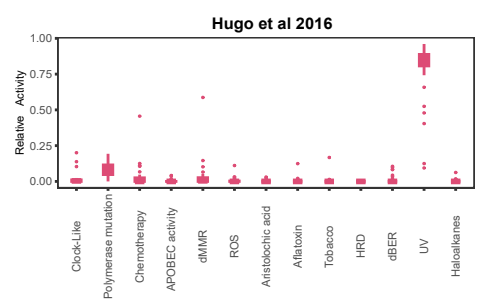**G**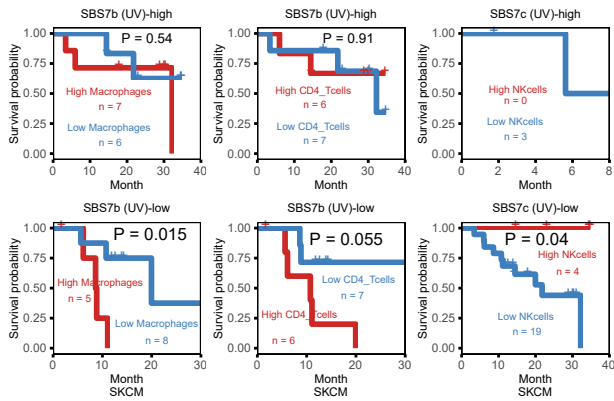**H**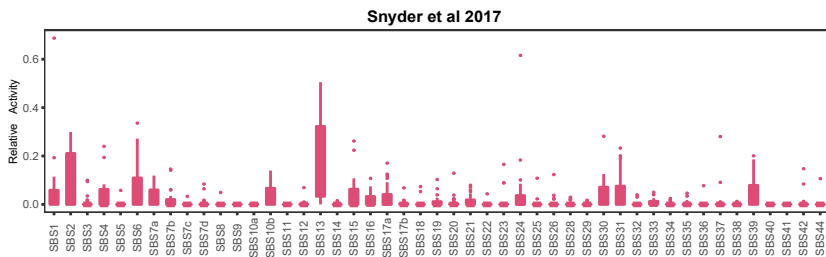**I**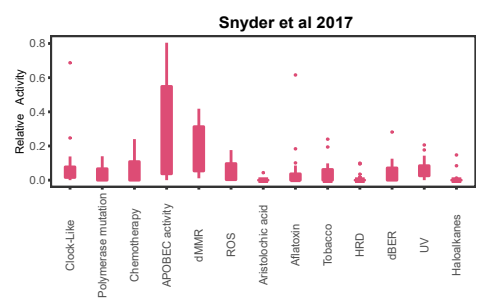**J**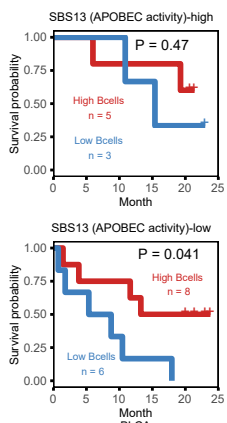**K**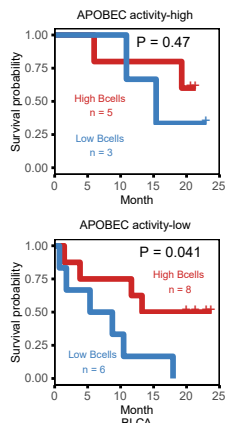

Supplement: Supplementary Figure 1 — Picture and validation of interplay between mutational signatures and the immune microenvironment in different cancer types. (A-C) Heatmaps showing association p values between SBS mutational signatures and TME cell types derived from approaches (A) Kassandra, (B) EPIC, and (C) quanTIseq. (D-F) Heatmaps showing association p values between etiology-associated mutational signatures and TME cell types derived from approaches (D) Kassandra, (E) EPIC, and (F) quanTIseq. Detailed data are available in Supplementary Tables 3 , 5 , 6 . [file DataSheet_1.zip › Supplementary_files/Supplementary_Fig6.pdf]
